# The Representation of Different Populations in Studies Assessing the Validity of Consumer Wearable Photoplethysmography-Based Measurements: Scoping Review

**DOI:** 10.2196/87324

**Published:** 2026-08-28

**Authors:** Rebecca M Schipper, Fatime Oumar Djibrillah, Meyke Roosink, Laura Winkens, Arlene John, Eric Hazebroek, Annemieke Witteveen, Agnes A M Berendsen

**Affiliations:** 1 Division of Human Nutrition and Health, Wageningen University & Research, P.O. Box 17, Wageningen, 6700 AA, The Netherlands, 31 655026289; 2 Department of Biomedical Signals and Systems, Faculty of Electrical Engineering, Mathematics and Computer Science, University of Twente, Enschede, The Netherlands; 3 TechMed Institute, University of Twente, Enschede, The Netherlands; 4 Faculty of Behavioral, Management and Social Sciences, University of Twente, Enschede, The Netherlands; 5 Consumption and Healthy Lifestyles Group, Wageningen University & Research, Wageningen, The Netherlands; 6 Vitalys Obesity Clinic, Rijnstate Hospital, Elst, The Netherlands

**Keywords:** wearable, PPG, photoplethysmography, representation, heart rate, heart rate variability, SpO_2_, blood pressure, respiration rate

## Abstract

**Background:**

Consumer wearables are increasingly being integrated into health research for data collection. Although they are attractive to use, the accuracy of their photoplethysmography (PPG)-based measurements can be influenced by user characteristics such as sex, age, BMI, and skin tone. However, our knowledge regarding the validity of these measurements in certain populations, such as those with darker skin tones, seems limited. This is concerning as uncorrected differences in measurement accuracy can lead to health disparities when consumer wearable measurements are used more frequently. A potential cause for the gap in our knowledge regarding consumer wearable validity is the underrepresentation of certain population groups in studies validating PPG-based consumer wearables.

**Objective:**

This scoping review aimed to map the representation of different sex, age, BMI, and skin tone groups in studies assessing the validity of PPG-based pulse rate, heart rate variability, blood pressure, peripheral blood oxygen saturation (SpO_2_), and respiratory rate measurements of consumer wearables.

**Methods:**

A literature search was conducted in Scopus, PubMed, and IEEE Xplore in July 2025. Papers were eligible if they assessed the validity of consumer wearable PPG-based measurements, expressed as the agreement with a reference method. From the included papers, the study population distribution of sex, age, BMI, and Fitzpatrick scale was extracted. To evaluate the representation, percentages of people in specific age, BMI, and skin tone groups were estimated based on reported means and SDs. The median percentage of participants in each population group, as well as the total percentage, is reported.

**Results:**

After the removal of duplicates, 734 papers were screened for eligibility. Following title and abstract screening, 238 papers remained, of which 186 passed full-text screening and were included in the review. Most of the studies (n=160) focused on pulse rate. Sex, age, BMI, and Fitzpatrick scale were reported by 179 (96.0%), 178 (96.0%), 101 (54.0%), and 35 (19.0%) out of 186 studies, respectively. While the median representation was 0% (IQR 0%-8%) for both older adults (>65 y) and individuals with obesity (BMI>30 kg/m^2^; IQR 0%-13%), aggregate participation across all studies was higher (1290/6367, 20.0% and 473/3428, 14.0%, respectively). Individuals with underweight (BMI<18.5 kg/m^2^) remained rare (median 3%, IQR 0%-7%), and the aggregate was 7.0% (225/3428). The median percentage of people with darker skin tones (Fitzpatrick type V and VI) participating in a study was 0%.

**Conclusions:**

Based on our results, it can be concluded that older adults and people with underweight, obesity, or darker skin tones are generally underrepresented in studies assessing the validity of consumer wearable PPG-based measurements. Future validation studies should focus more on the representativeness of the study population. This can be achieved by setting a benchmark for representativeness and including study population representatives during the study design process.

## Introduction

In 2012, the first commercial wrist-worn pulse rate sensor was released via a Kickstarter campaign [1]. It was developed for athletes to keep track of their pulse rate during training without having to wear a chest strap. Now, a little more than a decade later, the sensing functionalities of consumer wearables have expanded significantly. In addition to pulse rate, these wearables measure, among others, heart rate variability (HRV), peripheral blood oxygen saturation (SpO_2_), respiratory rate, blood pressure, skin temperature, and skin conductance. Using these measurements, composite measures such as stress and sleep quality scores are also derived. The added sensing functionalities have broadened the use of consumer wearables from a tool to enhance exercise performance in athletes to health and well-being monitoring for consumers. It is forecasted that the number of smartwatch users will reach 740.53 million in 2029 [2].

Not only have consumers embraced wearables, but the use of consumer wearables in health research is also increasing. A scoping review published in 2022, mapping the use of wearables in health research, identified 179 studies, of which 163 used a consumer wearable [3]. An increase in the number of studies using wearables published per year was observed, rising from 1 in 2013 to 21 in 2017 and doubling to 56 in 2020. In these studies, consumer wearables were implemented for various purposes, ranging from examining correlations between wearable-derived outcomes and other physiological outcomes to developing risk prediction models, disease screening, and monitoring.

The integration of consumer wearables into health research is attractive, as they enable continuous and remote measurements of diverse outcomes while being more comfortable for participants and often more affordable than medical-grade or research-grade sensors. It should be noted, however, that many consumer wearable measurements, including pulse rate, HRV, SpO_2_, blood pressure, and respiratory rate, are based on photoplethysmography (PPG). PPG is an optical measurement technique in which light is used to detect changes in blood volume in the skin microvascular bed [4]. The signal produced by this technique is sensitive to external factors, such as environmental light and movement, as well as user characteristics that influence the optical properties of the skin, such as sex, age, BMI, and skin tone [5-7]. For example, melanin in the skin, a determinant of skin tone, absorbs and scatters light, which influences the PPG signal quality [5]. The validity of PPG-based measurements thus varies between measurement conditions and users.

In the last few years, concerns have been raised about our limited knowledge regarding the validity of PPG-based consumer wearable measurements in certain population groups, such as people with darker skin tones or older individuals [8-10]. As manufacturers often do not report the validity of their consumer wearable measurements, researcher-initiated validation studies are being conducted to compare consumer wearable measurements with a reference method. Thus far, these validation studies have reported the accuracy of consumer wearable measurements under different conditions, such as during rest, physical activity, or sleep, in both controlled laboratory settings and free-living conditions [11,12]. However, the influence of characteristics such as sex, age, BMI, and skin tone on measurement validity remains largely unclear, and studies investigating this influence appear to be scarce [13-15]. If potential differences in measurement validity remain unidentified, these differences could contribute to variations in the accuracy of consumer wearable–based risk prediction, disease screening, and monitoring tools across populations. In turn, this may lead to an increase in health disparities—defined as preventable differences in the opportunity to achieve optimal health—as the integration of consumer wearables into health research and, eventually, health care progresses [8-10].

It is hypothesized that the underrepresentation of certain population groups in studies assessing the validity of PPG-based consumer wearable measurements contributes to the gaps in our knowledge about the validity of these measurements in different populations. To improve our understanding of these topics, the potential gaps in the representation of different population groups need to be identified and addressed. The aim of this scoping review is to systematically map the representation of different sex, age, BMI, and skin tone population groups in studies assessing the validity of PPG-based consumer wearable measurements, including pulse rate, HRV, blood pressure, blood oxygen saturation, and respiratory rate. Additionally, we will evaluate the representation of these different population groups to determine which groups require increased representation in future validation studies to enable the investigation of differences in measurement validity between population groups.

## Methods

### Study Design

This study was performed between May 2024 and August 2025 using the methodological framework for scoping reviews proposed by Arksey and O’Malley [16]. The registration and protocol for this review are available on the Open Science Framework (OSF) [17]. This review is reported according to the PRISMA (Preferred Reporting Items for Systematic Reviews and Meta-Analyses) extension for scoping reviews (PRISMA-ScR; Checklist 1) guidelines [18].

### Eligibility Criteria

Papers eligible for inclusion in this scoping review were those assessing the criterion validity, the agreement of a new measurement method with a criterion measure, of one or more commercial wearable sensors for measuring pulse rate, HRV, blood pressure, respiratory rate, and/or oxygen saturation in humans. The following exclusion criteria were applied:

- The wearable sensor that was validated is a chest strap, as chest straps are considered a criterion measure [19].
- The wearable sensor that was validated is not and was not at any point commercially available to consumers, as this scoping review focuses on consumer wearables only.
- The wearable sensor that was validated is not meant to be worn continuously during daily activities, as we consider this an important characteristic of a true consumer wearable device.
- The wearable measurement that was validated was measured using a method other than PPG, as this scoping review focuses on PPG-based measurements only.
- N=1 or case studies are excluded as this review has a focus on the distribution of characteristics within study populations.
- Animal studies are excluded, as this scoping review focuses on human participants.
- Reviews, editorials, opinion pieces, and protocols are excluded, as these are not primary research papers.
- No full text could be obtained, or insufficient information was provided in the full text. At least the sample size, the reference method used, and the name and brand of the wearable used should be mentioned to be able to verify if other exclusion criteria apply.
- Publication languages other than English are not considered, as English is the language in which all involved reviewers are proficient.

### Search Strategy

A literature search was conducted on June 11, 2024, in the databases Scopus, PubMed, and IEEE Xplore. To update the results before publication, the same search was repeated on July 16, 2025. The main concepts included in the search strategy were “PPG-based measurements,” “validity,” and “consumer wearable.” The keywords for these concepts were selected through pilot searches. The search strategy for all 3 databases can be found in Multimedia Appendix 1. Additionally, the reference lists of reviews returned by the database search were reviewed to identify additional papers.

### Screening and Selection

The database search results were uploaded to EndNote 20 (Clarivate) for duplicate removal, after which the remaining papers were uploaded to Rayyan for screening. Prior to initiating the official screening process, the screening guide was tested by 2 reviewers (RMS and FOD) during a pilot screening round with 10 papers. There were no conflicts between the 2 reviewers, and no adjustments were made to the screening guide. The reviewers, RMS and FOD, then proceeded to independently screen the papers for eligibility based on title and abstract, after which a full-text screening took place. Conflicts were resolved through discussion between the 2 reviewers.

### Data Charting

Extracted data included information on the type of publication, wearable, study context, recruitment and eligibility, and study population. A complete overview of the data items extracted is presented in Table 1. Most studies do not report how they assessed sex; therefore, it is often unclear whether they report sex or gender. For consistency within this review, we will refer to this characteristic as sex. For skin tone, it was decided to extract the Fitzpatrick scale as this is how most studies report skin tone [13], and as it is the measure recommended by several guidelines, including the US Food and Drug Administration (FDA) guideline on pulse oximeter evaluation, the guidelines for the evaluation of consumer wearable pulse rate validity published by the INTERLIVE network, and the ANSI/CTA (American National Standards Institute/Consumer Technology Association) standard [19-21].

The data charting form was created by RMS in Microsoft Excel. This form was used independently by 2 data extractors in a data charting pilot (RMS and FOD) with 10% of the included papers. For the main data items of interest, for example, study population characteristics, the percentage agreement between the 2 extractors was 95.5%. However, the agreement on the level of physical activity was low (40%). Therefore, additional guidelines for extracting the level of physical activity were added to the data extraction form, after which RMS proceeded with charting the data of the remaining papers independently.

**Table 1.** Extracted data items from 186 studies assessing the validity of consumer wearable photoplethysmography-based measurements.

Category	Extracted information
Publication information	Title, year of publication, first author, and DOI
Wearable	Brand, model, version of operating system, and intended wear location
Context	Study location, reference device, and level of physical activity
Recruitment and eligibility	Recruitment location, recruitment channels, and eligibility criteria
Study population	Sample size, distribution of sex, age, height, weight, BMI, and Fitzpatrick scale, and disease status

### Synthesis of Results

With the extracted data, the number of studies per PPG-based measurement, the number of wearable brands and models, studies per year, and per study location were determined. Additionally, the recruitment locations and eligibility criteria were summarized.

### Evaluating Representation

To provide an overview of the representation of population groups, 2 different approaches were applied. First, for the characteristic sex, the percentage of women in a study was extracted or calculated based on the number of women and the sample size. Based on this percentage, studies were categorized as equal distribution (at least 40% men or women), men-dominant or women-dominant (more than 60% men or women, respectively), men only, or women only. As none of the studies reported the participation of intersex individuals, this review can therefore only report the distribution of men and women.

Second, for age, weight, height, BMI, and Fitzpatrick scale, the weighted mean (SD) was calculated to capture the distribution of these characteristics in the included studies. Additionally, for age, BMI, and Fitzpatrick scale, the percentage of participants in commonly used groups of these characteristics was estimated to better visualize the representation of these different groups (Textbox 1) [22-24]. The percentages were estimated based on probability, using the mean and SD reported by the included studies [25]. First, the *z*-scores for the boundaries of each group were calculated using the provided mean and SD. Second, to estimate the probability of a value equal to or less than the boundary *z*-scores occurring, known as the cumulative distribution function, the *pnorm* function was used in RStudio (version 2023.6.1.524) with R (version 4.4.1). The percentage of participants in a group was then estimated by subtracting the cumulative distribution function of the lower boundaries from the cumulative distribution function of the upper boundaries. A threshold was applied to ensure that percentages lower than the contribution of 1 participant were rounded to 0. For age, the distribution was often truncated at 18 years of age, as studies only included adults. In these cases, the *ptruncnorm* function with a lower bound of 18 was used instead of the *pnorm* function.

Textbox 1. **Age, BMI, and skin tone categories used in a scoping review of 186 studies assessing the validity of consumer PPG-based measurements.**

**Age categories (years)**

- Children: <13
- Adolescents: 13‐17
- Adults: 18‐65
- Older adults: ≥65

**BMI categories (kg/m^2^)**

- Underweight: <18.5
- Normal weight: 18.5‐25
- Overweight: 25‐30
- Obesity: ≥30

**Skin tone categories**

- Fitzpatrick type I: always burns, never tans
- Fitzpatrick type II: usually burns, tans less than average
- Fitzpatrick type III: sometimes burns mildly, tans about average
- Fitzpatrick type IV: rarely burns, tans more than average
- Fitzpatrick type V: rarely burns, brown tone
- Fitzpatrick type VI: rarely burns, dark brown or black tone

To visualize the distribution of the representation across studies, the median (IQR) of the percentage of participants within each category was reported. To quantify the representation in all studies combined, the total number of participants within each category was calculated based on the estimated percentages. All plots and quantitative analyses were performed in RStudio (version 2023.6.1.524) with R (version 4.4.1).

To evaluate whether a population group was sufficiently represented, the median and total percentage of participants in this population group were compared to a predetermined benchmark [26]. For the purpose of this review, the selected benchmark is equal representation of all population groups. For example, for BMI with 4 groups, the percentage of participants in each BMI group should approach 25%. This benchmark was selected as equal representation of all population groups is desirable when statistically comparing group results.

## Results

### Study Selection

**Figure 1.**
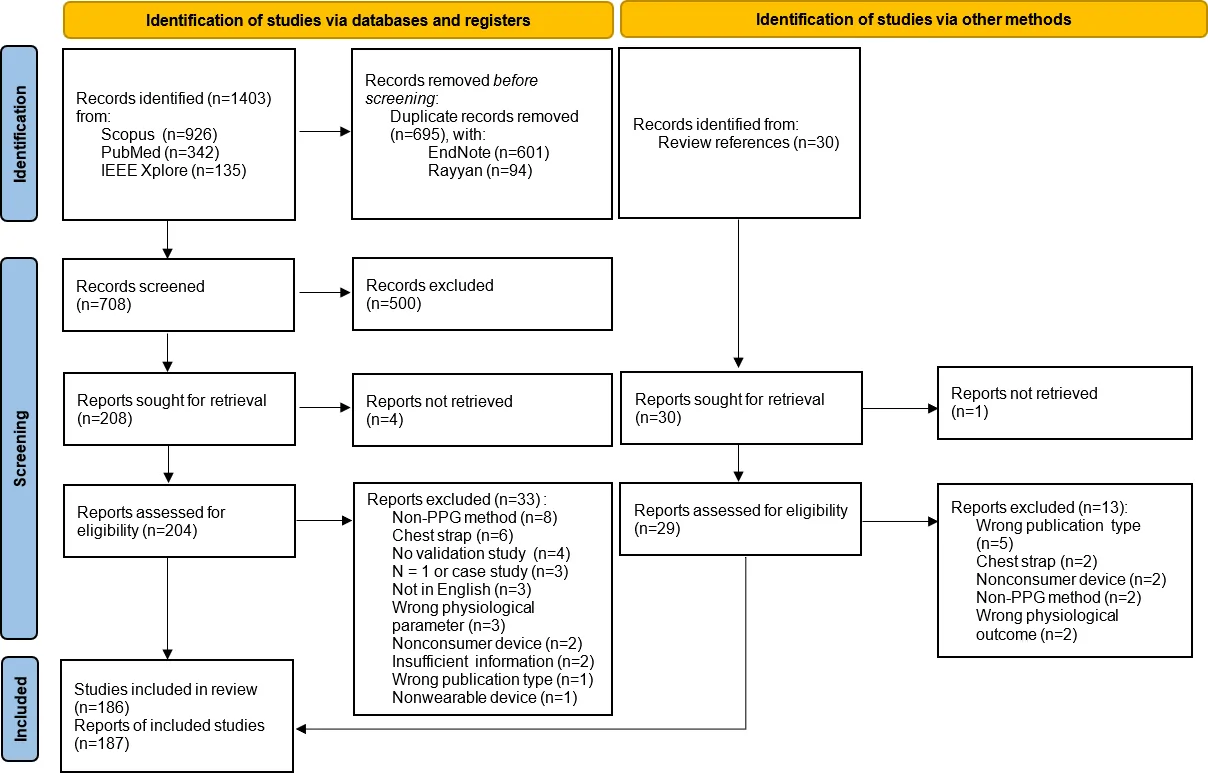
PRISMA flowchart. PPG: photoplethysmography; PRISMA: Preferred Reporting Items for Systematic Reviews and Meta-Analyses.

A total of 1403 papers were identified and extracted from PubMed, Scopus, and IEEE Xplore (Figure 1), of which 695 were identified as duplicates and removed, leaving 708 papers for title and abstract screening. Based on title and abstract screening, 500 papers were excluded, and an additional 5 papers were excluded because the full text could not be retrieved. Screening the reference lists of relevant reviews identified 29 additional papers for full-text screening. In the end, 233 papers were included in full-text screening. Based on full-text screening, 187 papers remained for inclusion, providing results from 186 individual studies.

### General Study Characteristics

**Figure 2.**
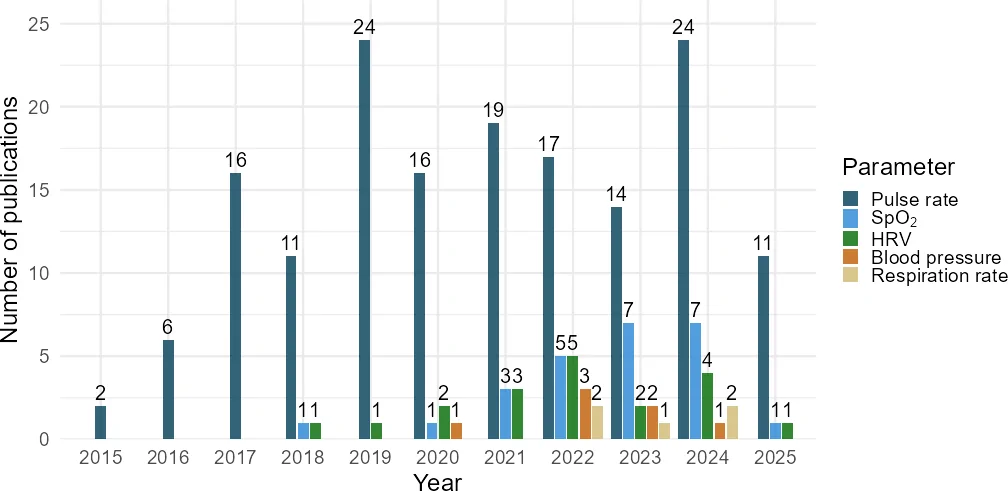
Number of studies validating consumer wearable measurements per photoplethysmography-based measurement per year, including studies published until July 16, 2025. HRV: heart rate variability; SpO_2_: oxygen saturation.

Of the 186 included studies (n=8786 participants), 160 (n=6461 participants) examined pulse rate, 25 (n=2053) oxygen saturation, 19 (n=887) HRV, 7 (n=326) blood pressure [27-33], and 5 (n=329) respiration rate [34-38]. The median (IQR) sample size was 30 (19-49) participants [19,39]. The publication year ranged from 2015 to 2025 (Figure 2), and studies were performed in 32 countries (Table 2). Across all studies, 33 wearable brands and 129 different models were used. Most of the examined wearables were wrist-worn (119 models), and some were worn on the upper arm (3 models), the finger (2 models) [40-44], or in the ear (5 models) [42,45-47]. Most studies (106 studies) validated 1 wearable device; the remaining 80 studies validated 2 to 7 wearable devices. Additional information about the study characteristics of studies for each examined PPG-based measurement can be found in Multimedia Appendix 2. The extracted data for each paper can be found in Multimedia Appendix 3.

**Table 2.** Number of studies validating consumer wearable photoplethysmography-based measurements by study location at the country level.

Country	Number of studies, n	References
United States	54	[6,32,39,42,43,45,47-94]
Italy	13	[27,30,35,95-104]
Germany	12	[34,36,46,105-113]
Australia	11	[41,114-123]
Canada	10	[124-133]
Republic of Korea	9	[37,134-141]
United Kingdom	9	[142-150]
Spain	8	[151-158]
Belgium	6	[28,38,159-162]
Brazil	6	[29,33,163-166]
Denmark	6	[167-172]
Switzerland	6	[44,173-177]
Finland	4	[40,178-180]
France	3	[181-184]
Ireland	3	[185-187]
Singapore	3	[188-190]
Austria	3	[100,191,192]
China	2	[31,193]
Czech Republic	2	[194,195]
Netherlands	2	[196,197]
Saudi Arabia	2	[198,199]
Taiwan	2	[200,201]
Turkey	2	[202,203]
India	1	[204]
Japan	1	[205]
Malaysia	1	[206]
New Zealand	1	[207]
Norway	1	[208]
Philippines	1	[209]
Poland	1	[210]
Sweden	1	[211]
Thailand	1	[212]

### Participant Eligibility and Recruitment

#### Eligibility Criteria

Of the 186 studies, 150 studies provided eligibility criteria for their study populations, and 69 (37.0%; n=3640 participants) studies stated that adults were eligible for participation. Moreover, there were 31 of 186 studies (7.0%; n=1001 participants) that only included younger adults, 10 (5.0%; n=684 participants) studies that only included children and/or adolescents, 4 (2.0%; n=97 participants) that only included older adults [38,92,163,185], and 1 (n=60 participants) that included children, adolescents, and adults [178]. Eight studies used BMI-based exclusion criteria, either excluding people who were underweight (3 studies) [78,116,188], people with overweight (1 study) [176], people with obesity (2 studies) [77,177], people with clinical obesity (BMI≥40 kg/m^2^, 1 study) [108], or both people who were underweight and overweight (1 study) [131].

#### Health Status

According to the description of the populations, out of 186 studies, most of them (n=137, 74.0%) included healthy populations. There were 47 (25.0%) studies that used the Physical Activity Readiness Questionnaire to screen participants for inclusion. Exclusion criteria for cardiovascular diseases (n=81, 44.0%), musculoskeletal injuries, diseases, or disabilities (n=46, 25.0%), and pregnancy (n=11, 6.0%) were common. Of 186 studies, 49 (26.0%; n=4921 participants) were performed on patient populations with various conditions. These conditions included cardiovascular diseases (21 studies), respiratory diseases (8 studies) [38,49,53,82,101,166,169,202], surgeries (4 studies) [33,36,98,108], disabilities (3 studies) [167,197,208], breast cancer (1 study) [92], COVID-19 (1 study) [123], chronic musculoskeletal pain (1 study) [211], and cystic fibrosis (1 study) [116].

#### Recruitment Location

Of the 186 studies, 91 (49.0%) studies provided information on the recruitment location; 46 (25.0%) studies recruited participants from a hospital, and 26 (14.0%) studies recruited participants from a university or research group exclusively. Other recruitment settings included local communities and associations (n=9, 5.0%) [55,80,92,132,136,140,142,185,187], patient organizations (n=3, 2.0%) [167,197,208], professional environments such as the military (n=3, 2.0%) [121,174,176], and a physical activity challenge (n=1, 1.0%) [188]. One study included participants from a previous trial [148].

### Reporting of Study Population Characteristics

**Figure 3.**
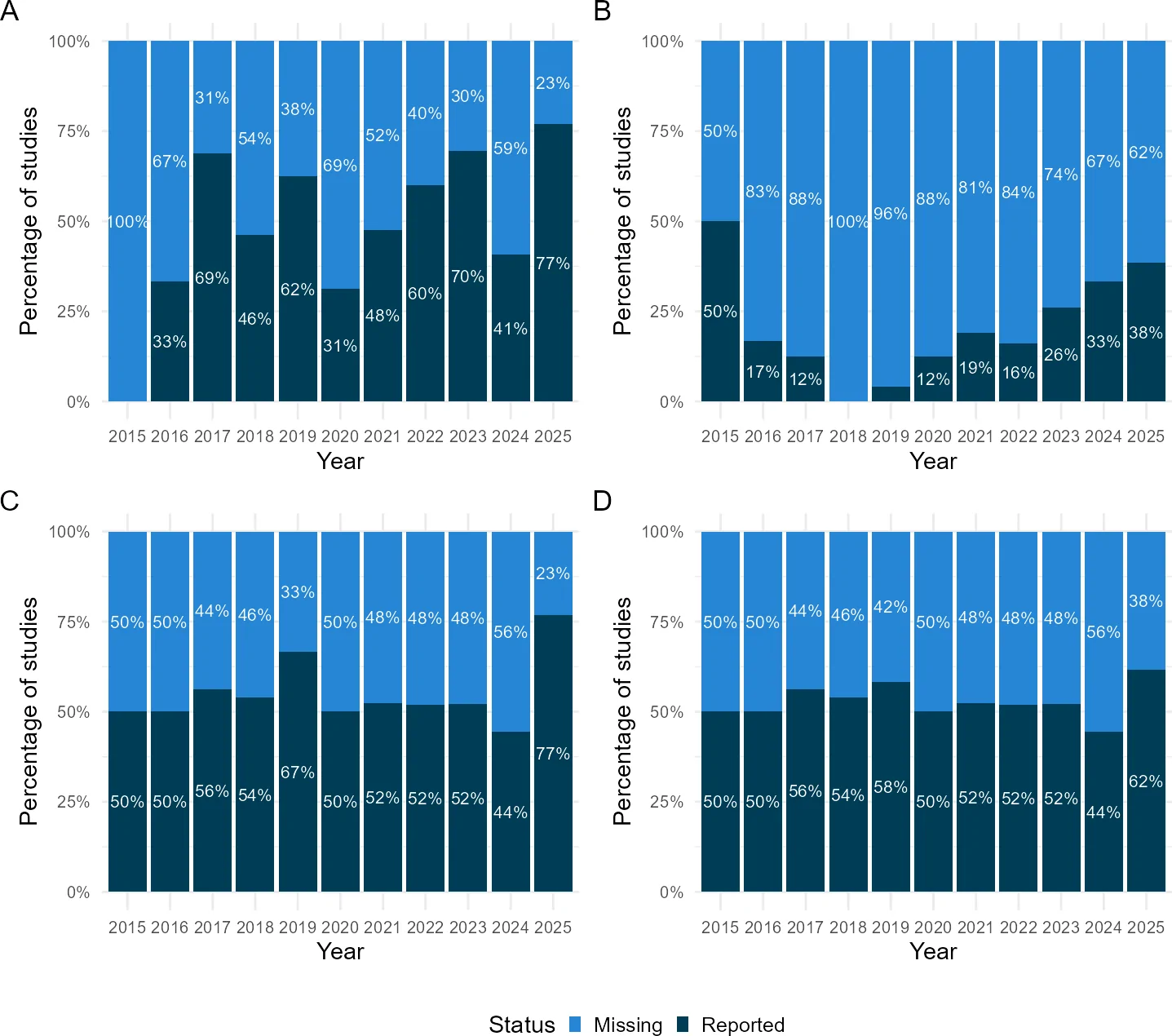
The percentage of consumer wearable photoplethysmography-based measurement validation studies per year reporting BMI (A), Fitzpatrick scale (B), weight (C), and height (D).

In the 186 studies reviewed, the most reported study population characteristics were age and sex (n=178, 96.0%, and n=179, 96.0%, respectively), followed by BMI (n=101, 54.0%), weight (n=102, 55.0%), height (n=98, 53.0%), and finally the Fitzpatrick scale (n=35, 19.0%; Table 3). Over time, the percentage of studies reporting weight and height remained stable at ~50% (Figure 3). The percentage of studies reporting BMI fluctuated over time, with a minimum of 0% (0/2) in 2015 and a maximum of 77.0% (10/13) in 2025. An upward trend in the reporting of the Fitzpatrick scale was observed from 2019 to 2025 (1/24, 4.0% to 5/13, 38.0%).

**Table 3.** Number (%) of consumer wearable photoplethysmography (PPG)-based measurement validation studies reporting sex, age, BMI, weight, height, and Fitzpatrick scale.

Characteristics	All	PR^a^	HRV^b^	SpO_2_^c^	BP^d^	RR^e^
Studies, N	186	160	19	25	7	5
Sex, n (%)	179 (96.0)	157 (99.0)	16 (84.0)	23 (92)	7 (100)	5 (100)
Age, n (%)	178 (96.0)	156 (98.0)	16 (84.0)	22 (88)	7 (100)	4 (80)
BMI, n (%)	101 (54.0)	87 (54.0)	7 (37.0)	13 (52)	3 (43.0)	4 (80)
Weight, n (%)	102 (55.0)	93 (58.0)	6 (32.0)	12 (48)	1 (14.0)	1 (20)
Height, n (%)	98 (53.0)	89 (56.0)	5 (26.0)	12 (48)	1 (14.0)	1 (20)
Fitzpatrick scale, n (%)	35 (19.0)	31 (19.0)	3 (16.0)	9 (36)	1 (14.0)	2 (40)
All, n (%)	15 (8.0)	14 (9.0)	2 (11.0)	3 (12)	0 (0)	1 (20)

a PR: pulse rate.

b HRV: heart rate variability.

c SpO_2_: oxygen saturation.

d BP: blood pressure.

e RR: respiratory rate.

Age, BMI, weight, and height were mostly reported as mean or median combined with an SD, SE, or IQR (age: 158/178, 89.0%; BMI: 98/101, 97.0%; weight: 98/102, 96.0%; height: 94/98, 96.0%). A lower percentage of studies additionally reported the range (age: 44/178, 25.0%; BMI: 25/101, 25.0%; weight: 25/102, 25.0%; height: 23/98, 23.0%). Reporting of the Fitzpatrick scale was highly variable, with studies either reporting a combination of mean, median, SD, or IQR (12/35, 34.0%), the percentage of participants in each level or in a combination of at least 2 levels (6/35, 17.0%, for both), the range (5/35, 14.0%), or a combination of these options (6/35, 17.0%).

### Representation of Study Population Characteristics

#### Representation of Men and Women

In 179 studies (n=8203 participants), the percentage of women in a study was 43±19%. Most of the studies (102 studies, n=4570 participants) included an approximately equal percentage of men and women. There were 46 men-dominant studies (n=2637 participants), 17 women-dominant studies (n=688 participants), 11 men-only (n=277 participants) [59,121,135,146,157,164,167,174,199], and 3 women-only (n=31 participants) studies [35,136,213].

#### Representation of Age Groups

The weighted mean (SD) of age across 149 studies (n=6367) reporting these statistics was 44 (11.9) years. The lowest reported age was 1.2 months [127], and the highest reported age was 94 years [126,202]. In the 112 studies (n=5088), which did not exclude older adults and reported the mean (SD), the median (IQR) percentage of older adults in a study was 0% (0%-8%). The total estimated number of older adults participating across the 149 studies that reported the mean (SD) was 1290 of 6367 participants (20.0%). The results of each examined PPG-based measurement separately are presented in Table 4 and Multimedia Appendix 4. Of these 1290 estimated older adults, 1247 (97.0%) participated in studies with a focus on patient populations. From 2021 to 2024, the total percentage of older adults participating each year increased from 23% to 38% (Multimedia Appendix 5).

**Table 4.** Percentage (median, IQR) of older adults and participants belonging to BMI and skin tone groups in consumer wearable photoplethysmography-based measurement validation studies.

Participant characteristics	PR^a^^,^^b^, median (IQR)	HRV^c^^,^^d^, median (IQR)	BP^e^^,^^f^, median (IQR)	SpO_2_^g^^,^^h^, median (IQR)	RR^i^^,^^j^, median (IQR)
Age					
Older adults	0 (0‐0)	0 (0‐0)	11 (2-28)	6 (0‐27)	11 (6-14)
BMI					
Underweight	4 (0‐7)	6 (3-7)	4 (2-6)	3 (0‐3)	3 (1-3)
Normal weight	58 (41-72)	42 (36-46)	23 (30-37)	34 (26-50)	37 (26-68)
Overweight	31 (17-36)	34 (27-36)	34 (30-37)	40 (28-41)	27 (13-35)
Obesity	0 (0‐12)	13 (9-19)	37 (36-38)	20 (0‐33)	15 (8-34)
Fitzpatrick scale					
I	15 (7-24)	18 (16-20)	—^k^	15 (3-21)	—
II	35 (19-45)	32 (24-41)	—	34 (26-46)	—
III	25 (14-30)	20 (20,21)	—	21 (15-30)	—
IV	9 (3-21)	9 (4-13)	—	10 (2-18)	—
V	0 (0‐9)	9 (4-13)	—	1 (0‐3)	—
VI	0 (0‐0)	9 (5-14)	—	0 (0‐2)	—

a PR: pulse rate.

b Age: n=132; BMI: n=78; Fitzpatrick scale: n=16.

c HRV: heart rate variability.

d Age: n=12; BMI: n=5; Fitzpatrick scale: n=2.

e BP: blood pressure.

f Age: n=6; BMI: n=2.

g SpO_2_: peripheral blood oxygen saturation.

h Age: n=16; BMI: n=8; Fitzpatrick scale: n=6.

i RR: respiratory rate.

j Age: n=5; BMI: n=3.

k Not applicable.

#### Representation of BMI Categories

**Figure 4.**
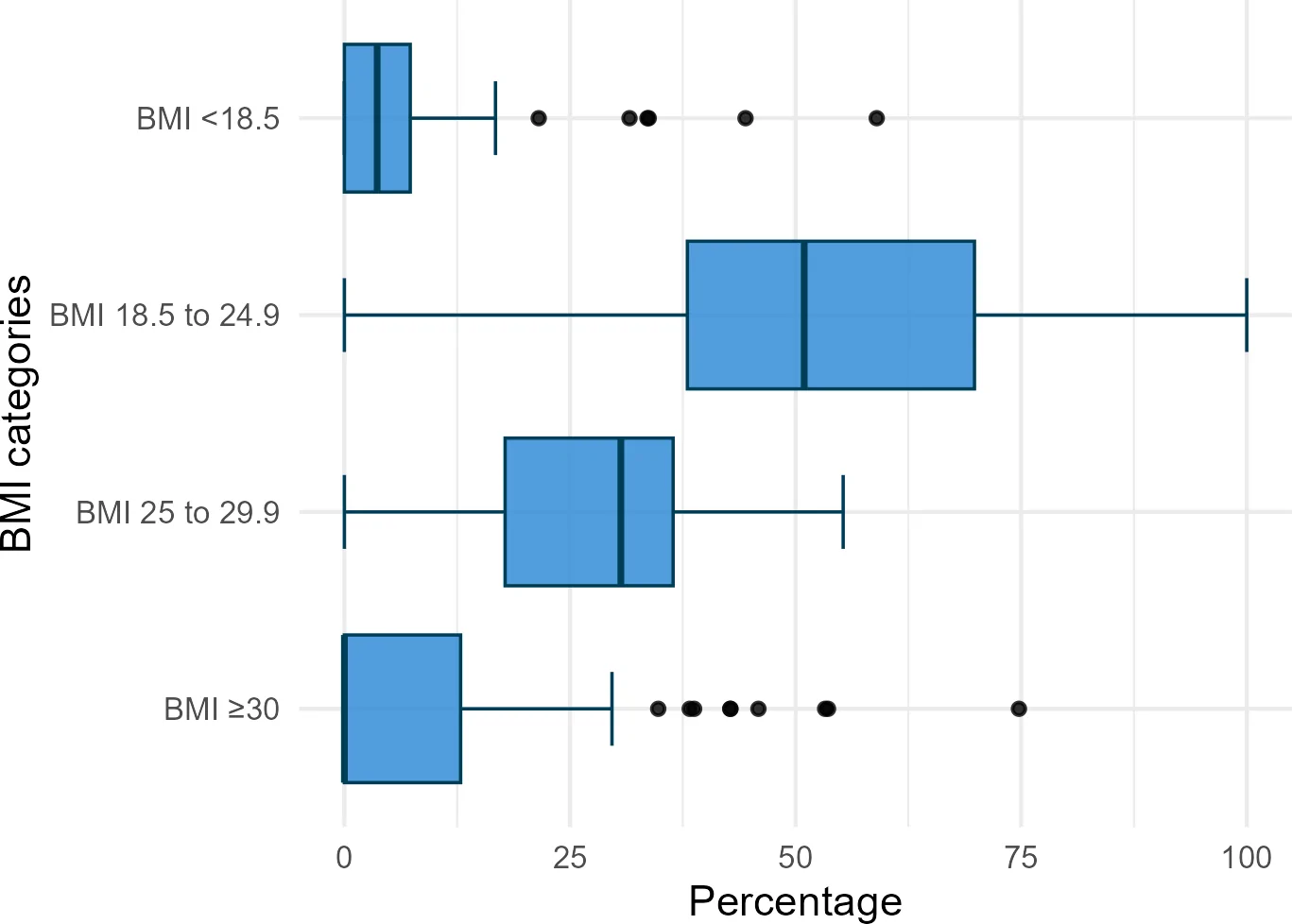
Boxplot of the distribution of the estimated percentage of participants in 4 BMI (kg/m^2^) categories in 89 studies (n=3428 participants) validating consumer wearable photoplethysmography-based measurements.

Based on 89 studies (n=3428), the weighted mean (SD) of BMI was 24.8 (4.2) kg/m^2^, with BMIs ranging from 16.3 [118] to 52.0 kg/m^2^ [34]. In these studies, the median (IQR) percentage of people with underweight, normal weight, overweight, and obesity in a study was 3% (0%-7%), 51% (38%-70%), 31% (18%-37%), and 0% (0%-13%), respectively (Figure 4). Of the 3428 participants, it was estimated that 225 (7.0%) were underweight, 1602 (47.0%) had normal weight, 1048 (31.0%) were overweight, and 473 (14.0%) had obesity. Of the 473 estimated participants with obesity, 364 (77.0%) participated in studies that focused on patient populations. The representation of people with obesity increased over time from 4% in 2016 to 22% in 2022, after which it decreased to 15% in both 2023 and 2024 (Multimedia Appendix 5).

#### Representation of Fitzpatrick Scale Categories

**Figure 5.**
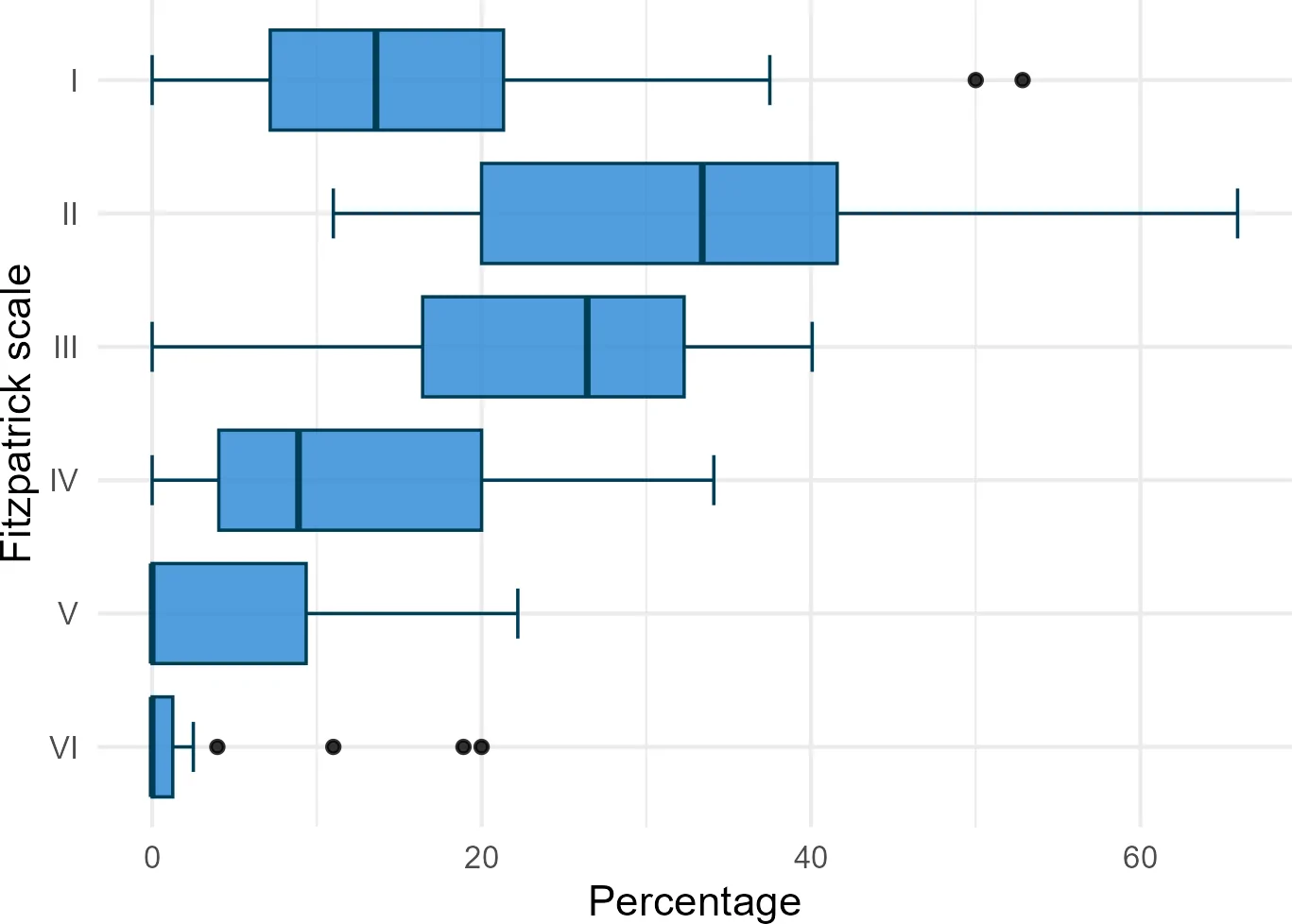
Boxplot of the distribution of the estimated percentage of participants in the Fitzpatrick scale categories in 19 studies (n=876 participants) validating consumer wearable photoplethysmography-based measurements.

In the 19 studies (n=876) that reported the mean (SD) or percentage of participants in each Fitzpatrick scale category, the majority of participants fell in type II (median 33%, IQR 20%-42%), while the minority were in types V and VI (median 0%, IQR 0%-9% and median 0%, IQR 0%-1%, respectively; Figure 5). Over the years, the representation of the different categories fluctuated, with no clear trend toward increasing or decreasing representation of any category (Multimedia Appendix 5).

## Discussion

### Principal Findings

This scoping review aimed to provide an overview of the representation of different sex, age, BMI, and skin tone groups in studies assessing the validity of PPG-based consumer wearable measurements. Based on the inclusion of 186 studies, our results indicate that the representation of the different population groups is not equal. In particular, older adults, people who were underweight, people living with obesity, and people with darker skin tones (Fitzpatrick type V or VI) are underrepresented compared to other groups. When interpreting our findings, it is important to consider the following points of discussion: (1) the reporting of user characteristics by the included studies; (2) the benchmark used for evaluating representation; and (3) the relevance of evaluating both study-level and total representation.

First, the results of this study revealed that a large percentage of studies assessing the validity of consumer wearables’ PPG-based measurements did not report BMI and skin tone. Not only does this limit our ability to fully evaluate the representation of these characteristics in this review, but it also indicates a critical gap in the available evidence. The nonreporting of these characteristics provides a barrier to investigating the influence of these characteristics on measurement validity. To increase the reporting of study population characteristics like BMI and skin tone, reporting guidelines for consumer wearable validation studies, such as those provided by the INTERLIVE network and the ANSI/CTA, could help. These reporting guidelines recommend the reporting of study populations’ sex, age, weight, height, BMI, and skin tone distribution for studies validating pulse rate measurements and could be extended toward other PPG-based validation studies [19,20]. To truly achieve improved reporting, guidelines like those from the INTERLIVE network should additionally be endorsed by journals that publish validation studies [214,215].

Second, the benchmark used to evaluate representation impacts what is considered sufficient representation and underrepresentation. For example, using equal representation as a benchmark, we consider people with underweight, with a median representation of 3%, to be underrepresented. However, using the prevalence of underweight in the general population, namely 1.6%, as a benchmark, the representation of people who are underweight would be considered sufficient [216,217]. The most suitable benchmark depends on the study’s aim. When the aim is to investigate the influence of sex, age, BMI, and skin tone on wearable measurement validity, equal representation of these groups is a suitable benchmark. However, if a validation study aims to reflect performance in a specific target population or the general population, then equal representation of all groups would not be a suitable benchmark. For this purpose, the prevalence of sex, age, BMI, and skin tone groups in the target or general population should be used as a benchmark. The Clinical Trial Diversity Rating framework, developed by the Institute for Clinical and Economic Review, can then be used to evaluate if this benchmark is sufficiently met [26]. Selecting a suitable benchmark is an important step toward achieving study population representativeness.

Third, in this review, we evaluated representation at the level of individual studies as well as total representation. There are population groups that had low representation at both levels, such as children and adolescents, people who are underweight, and people with darker skin tones (Fitzpatrick types V and VI). However, for older adults and people with obesity, although the representation at the study level was very low, the overall representation was higher. This discrepancy can occur if there is a particular subset of studies in which a group is highly represented, while in other studies, the representation is low. For example, in this scoping review, there was a subset of studies with large sample sizes conducted in patient populations in which older adults were highly represented, while in the remaining studies with smaller sample sizes, almost no older adults participated. In these validation studies, the older adults represented are thus mostly older adults living with disease, who might not be an accurate representation of healthy older adults. This pattern was only observed because we evaluated representation both at the level of individual studies and at the overall level, indicating the relevance of evaluating both levels. In addition to evaluating representation at these 2 levels, it would have been valuable to examine the representation of demographic characteristics intersectionally. Based on the information provided by the included studies, this was not feasible. In reality, the 4 characteristics investigated in this review coexist in one individual, and their cumulative effect is what influences signal quality. An older individual with obesity and dark skin, for example, could present an “intersectional stress test” that current algorithms frequently fail. This necessitates stratified sampling and reporting in future validation studies.

### Increasing the Representation of Underrepresented Population Groups

There are 2 explanations for the underrepresentation observed in this review. The first cause is the active exclusion of some population groups through exclusion criteria. For example, many of the validation studies investigating pulse rate measurement validity included in this review had a vigorous exercise component, which can be harmful to some population groups, including older adults. Likely due to this ethical consideration, 31 studies excluded older adults. The second cause is barriers to participation related to other elements of the study. These elements included the research question, the composition of the research team, the recruitment methods, the complexity of research processes, and the required investment of time and resources from the participants [218]. For example, none of the studies had skin tone-based eligibility criteria, yet the representation of people with darker skin tones was very low, which suggests that some other study elements could have prevented their participation.

Irrespective of the cause, the underrepresentation of older adults, people who are underweight, people with obesity, and people with darker skin tones currently provides a barrier for us to gain a better understanding of the differences in measurement validity between population groups. If there are relevant differences in consumer wearable measurement validity between these groups, as suspected, we need to take them into account when analyzing consumer wearable measurement data and interpreting the results. If not, differences in the accuracy of consumer wearable-based risk prediction, disease screening, and monitoring tools between populations may be created, exacerbating already existing health disparities as the implementation of these tools progresses. Additionally, a reduced accuracy of these tools in older adults and people with obesity poses a public health risk, as these tools can be used to screen for conditions that are more prevalent in these population groups (eg, atrial fibrillation and sleep apnea) [219]. Increasing the representation of the “missing” population groups in consumer wearable validation studies is therefore vital. To increase the representation of these groups, a suitable benchmark for representation should be selected during the study design process, and the representativeness of the study population should be evaluated based on that benchmark throughout the entire study. Ideally, this evaluation should not only be conducted by the research team but also by funding agencies, journals, peer reviewers, and other stakeholders in the research process. Additionally, researchers should involve their study populations in the study design process to reduce barriers to participation.

### Strengths and Limitations

This scoping review is the first to provide a systematic overview of the representation of sex, age, BMI, and skin tone groups in consumer wearable validation studies. Through an extensive search in 3 electronic bibliographic databases and the reference lists of 58 reviews, we were able to include 186 relevant papers. To ensure the transparency of our methods, the entire review process was guided by a protocol that is openly available on OSF. Nevertheless, this review has some limitations that need to be considered.

The representation of age, BMI, and skin tone groups was estimated based on the reported mean (SD), assuming a normal distribution. Though this approach allowed us to visualize the representation of these population groups, there is likely some overestimation or underestimation in these percentages. We hypothesize, based on our results, that the distribution of age, BMI, and skin tone is more likely to be skewed to the right in the included validation studies. An overestimation of the percentage of older adults, people with obesity, and people with darker skin tones in this review is therefore more likely. This suggests that the true representation of these population groups could be even lower than the percentages presented in this review. This is important to take into account when interpreting our findings, as is the fact that there is likely still variation in the representation within the categories applied in this review. Especially within the category of adults, which includes both young adults (18‐40 y) and middle-aged adults (41‐64 y), the high representation of young adults might mask the underrepresentation of middle-aged adults. Similarly, as no breakdown of the representation of the demographic groups per brand or model is reported in this review, it is important to note that there may be differences in the adequacy of representation between different devices, which are not reported in this review.

Another limitation of this review is the use of the Fitzpatrick scale to assess the representation of skin tone. The Fitzpatrick scale was included in this review because alternative measures are currently limited, and this scale is recommended by current reporting guidelines. However, it does not accurately depict the true representation of different skin tones with different melanin content, which truly impacts the PPG signal by absorbing and scattering the light [5]. Therefore, we recommend that future validation studies consider the use of melanometry, a method using reflected light that has been suggested as an objective quantitative measurement of skin melanin [220]. Future studies should also consider using arterial blood gas as a reference standard for measuring SpO_2_ instead of finger pulse oximeters. It is a critical limitation that many included studies used finger pulse oximeters, as these devices also exhibit pigment-related bias. Comparing wearables against them, rather than arterial blood gas, may obscure the true extent of skin tone inaccuracies. Additionally, it should be noted that BMI, like the Fitzpatrick scale, is a crude proxy for what it aims to measure, namely, adiposity. Although BMI is convenient for reporting and provides a general overview of the representation of different body sizes, future validation studies focusing on the mechanistic determinants of the validity of PPG-based measurements should consider measuring body composition.

Additionally, it is important to consider that this review only included studies that were published in English. This could have resulted in an underrepresentation of studies conducted in Asia, Africa, and South America, thereby reducing the representation of darker skin tones observed in this review. During the screening of the search results, only 3 studies were excluded based on the language criterion. This suggests that our search strategy, in general, was not very effective in retrieving non-English validation studies. If a similar review is performed in the future, we therefore recommend investigating methods to improve the retrieval of non-English studies, such as including local scientific literature databases.

### Conclusions

With this scoping review, we aimed to identify underrepresented population groups by mapping the representation of sex, age, BMI, and skin tone groups in 186 consumer wearable PPG-based measurement validation studies. Based on the results, we conclude that older adults, people with underweight or obesity, and people with darker skin tones are generally strongly underrepresented in consumer wearable validation studies. Researchers making use of PPG-based consumer wearable measurements in their studies should, therefore, be aware of the limited knowledge of the measurement validity in these population groups and take this uncertainty into account when interpreting their findings. To avoid the creation of further health disparities, there should be more focus on the representativeness of consumer wearable validation study populations. More transparent reporting of study population characteristics and the use of benchmarks for representation will allow researchers and other stakeholders in the research process to evaluate the representativeness of a study population. To achieve study populations that properly represent the target population, representatives from the target population should be included in the study design process.

## Supplementary material

10.2196/87324 Multimedia Appendix 1 Full search queries for each database.

10.2196/87324 Multimedia Appendix 2 Study characteristics per photoplethysmography-based measurement.

10.2196/87324 Multimedia Appendix 3 Study results per source. Sources are presented in alphabetical order.

10.2196/87324 Multimedia Appendix 4 Representation of the different population groups for each photoplethysmography-based measurement.

10.2196/87324 Multimedia Appendix 5 Representation of age, BMI, and Fitzpatrick scale categories over time.

10.2196/87324 Checklist 1 PRISMA-ScR checklist.

## Abbreviations

ANSI: American National Standards Institute
CTA: Consumer Technology Association
HRV: heart rate variability
PPG: photoplethysmography
PRISMA: Preferred Reporting Items for Systematic Reviews and Meta-Analyses
PRISMA-ScR: PRISMA extension for scoping review
SpO_2_: oxygen saturation
