# Supplementary material for: The Representation of Different Populations in Studies Assessing the Validity of Consumer Wearable Photoplethysmography-Based Measurements: Scoping Review
Source: JMIR Mhealth Uhealth. 2026 Aug 28;14:e87324. doi: 10.2196/87324 (PMC13524368; doi:10.2196/87324)
Supplement: Supplementary file 2 [file mhealth-v14-e87324-s001.docx]

## Multimedia Appendix 1: Search Query for each database

### Scopus

| # | Query |
| --- | --- |
| 1 | TITLE-ABS-KEY ("heart rate" OR "pulse rate" OR "cardiac rate" OR "heartbeat" OR "blood pressure" OR "pulse pressure" OR "systolic pressure" OR "diastolic pressure" OR "respiratory rate" OR "respiration rate" OR "oxygen saturation" OR "blood oxygen level" OR spo2) |
| 2 | TITLE-ABS-KEY ( valid* OR accura* OR "bland-altman" ) |
| 3 | TITLE-ABS-KEY ("commercial wearable" OR "commercial smartwatch" OR "commercially available wearable" OR "consumer wearable" OR fitbit OR "apple watch" OR garmin OR "galaxy watch" OR "polar watch" OR "polar vantage" OR "polar ignite" OR "polar pacer" OR "polar unite" OR "polar grit" OR "huawei watch" OR "omron heartguide" OR oura) |
| 4 | 1 AND 2 AND 3 |

### PubMed

| # | Query |
| --- | --- |
| 1 | "heart rate"[MeSH Terms] |
| 2 | "heart rate"[Title/Abstract] OR "pulse rate"[Title/Abstract] OR “cardiac rate”[Title/Abstract] OR "heartbeat"[Title/Abstract] |
| 3 | 1 OR 2 |
| 4 | “blood pressure”[MeSH Terms] |
| 5 | "blood pressure"[Title/Abstract] OR "pulse pressure"[Title/Abstract] OR "systolic pressure"[Title/Abstract] OR "diastolic pressure"[Title/Abstract] |
| 6 | 4 OR 5 |
| 7 | "respiratory rate"[MeSH Terms] |
| 8 | "respiratory rate"[Title/Abstract] OR "respiration rate"[Title/Abstract] |
| 9 | 7 OR 8 |
| 10 | "oxygen saturation"[MeSH Terms] |
| 11 | "oxygen saturation"[Title/Abstract] OR "blood oxygen level"[Title/Abstract] OR "spo2"[Title/Abstract] |
| 12 | 10 OR 11 |
| 13 | 3 OR 6 OR 9 OR 12 |
| 14 | "validity"[Title/Abstract] OR "valid"[Title/Abstract] OR "validated"[Title/Abstract] OR "validation"[Title/Abstract] OR "accurate"[Title/Abstract] OR "accuracy"[Title/Abstract] OR "accurately"[Title/Abstract] OR "bland-altman"[Title/Abstract] |
| 15 | "commercial wearable"[Title/Abstract] OR "commercial smartwatch"[Title/Abstract] OR "fitbit"[Title/Abstract] OR "apple watch"[Title/Abstract] OR "garmin"[Title/Abstract] OR "galaxy watch"[Title/Abstract] OR "polar watch"[Title/Abstract] OR "polar vantage"[Title/Abstract] OR "polar ignite"[Title/Abstract:~0] OR "polar pacer"[Title/Abstract:~0] OR "polar unite"[Title/Abstract:~0] OR "polar grit"[Title/Abstract] OR "huawei watch"[Title/Abstract] OR "omron heartguide"[Title/Abstract] OR "oura"[Title/Abstract] |
| 16 | 13 AND 14 AND 15 |

### IEEE xplore search strategy

| # | Query |
| --- | --- |
| 1 | "All Metadata":"heart rate" OR "All Metadata":"pulse rate" OR "All Metadata":"heartbeat" OR "All Metadata":"blood pressure" OR "All Metadata":"respiratory rate" OR "All Metadata":"respiration rate" OR "All Metadata":"oxygen saturation" OR "All Metadata":"blood oxygen level" OR "All Metadata":spo2 |
| 2 | "All Metadata":valid* OR "All Metadata":accura* OR "All Metadata":"bland-altman" |
| 3 | "All Metadata":"commercial wearable" OR "All Metadata":"commercial smartwatch" OR "All Metadata":"commercially available wearable" OR "All Metadata":"consumer wearable" OR "All Metadata":fitbit OR "All Metadata":"apple watch" OR "All Metadata":garmin OR "All Metadata":"galaxy watch" OR "All Metadata":"polar watch" OR "All Metadata":"polar vantage" OR "All Metadata":"polar ignite" OR "All Metadata":"polar pacer" OR "All Metadata":"polar unite" OR "All Metadata":"polar grit" OR "All Metadata":"huawei watch" OR "All Metadata":"omron heartguide" OR "All Metadata":oura |
| 4 | 1 AND 2 AND 3 |

### Updated search on July 16^th^ 2025

When the search was updated on July 16^th^ 2025, the search queries stated in this Multimedia Appendix were used. However, a date filter was added in all three databases to only retrieve records that were updated or added since the search on June 11^th^ 2024. In Scopus, the LOAD-DATE field code was used, in PubMed, the [dp] field code was used, and in IEEE Xplore, the “date Added to IEEE Xplore” field in the advanced search was used.
