# Supplementary material for: The Representation of Different Populations in Studies Assessing the Validity of Consumer Wearable Photoplethysmography-Based Measurements: Scoping Review
Source: JMIR Mhealth Uhealth. 2026 Aug 28;14:e87324. doi: 10.2196/87324 (PMC13524368; doi:10.2196/87324)
Supplement: Supplementary file 3 [file mhealth-v14-e87324-s002.docx]

## Multimedia Appendix 2: General characteristics per PPG-based measurement

#### Pulse rate

In the 160 studies focusing on pulse rate, a total of 125 different wearable models were used. The Fitbit Charge HR (28 studies), the first Apple Watch (21 studies) and the Fitbit Charge 2 (16 studies) were the most used models. As reference methods, a multi-lead ECG (85 studies), a chest strap like the Polar H7 or H10 (70 studies), a pulse oximeter (11 studies), a sphygmomanometer (1 study)[1] or an automatic oscillometer (1 study) was used[2]. The wearables have been validated during sleep (15 studies), rest (114 studies), light (41 studies), moderate (95 studies), and vigorous activity (84 studies).

#### Oxygen saturation

The 25 studies that focused on SpO_2_ used 17 different wearable models. The Apple Watch S6 (7 studies)[3-9], Apple Watch S7 (5 studies)[10-14], and the Withings ScanWatch (4 studies)[10, 11, 13, 15] were used most often. The reference method used by all studies was pulse oximetry. Two studies additionally used arterial blood gas as a criterion measure[3, 16]. Most studies assess SpO_2_ during rest (21 studies), and 4 studies also assessed it during sleep[10, 17-20], and 3 studies assessed after exercise[16, 21, 22].

#### Heart rate variability

In the 19 studies with a focus on HRV, 27 different wearable models were used. The most used models were the Oura Ring (3 studies)[23-25] and the Apple Watch (2 studies)[26, 27]. The remaining 27 models were each used in a singular study. As the reference method, 14 studies used a multi-lead ECG, while 5 studies used a chest strap [27-31]. Twelve studies included measurements during rest, 6 studies during sleep [18, 23-25, 28, 32], and 8 studies during exercise (5 light, 2 moderate, 1 vigorous).

#### Blood pressure

Seven studies focused on blood pressure measurement validity. In these studies, 7 different wearable models were used. The most used model was the Samsung Galaxy Watch 3, which was used in 2 studies. As the reference method, 5 studies used an automatic oscillometer [1, 2, 33-35], and 2 studies used both an automatic oscillometer and a manual sphygmomanometer[36, 37]. All seven studies assessed validity during rest, but two studies also assessed validity directly after light and moderate[37], and moderate and vigorous exercise[1].

#### Respiratory rate

In the 5 studies that focused on respiratory rate, 9 different wearable models were used. None of these was tested in multiple studies. A chest strap (2 studies)[30, 38], polysomnography (2 studies)[18, 19], and a multi-lead ECG and pulse oximeter (1 study) [10] were used as reference methods. Two studies measure respiratory rate during rest[10, 38], three during sleep[10, 18, 19], and one during free-living activities during the day[30].

## References

1.	Antognoli L, Panni L, Cosoli G, Scalise L. Metrological Characterization of Commercial Smartwatches: are these Sensors Suitable for the Assessment of Well-being? Università Politecnica Delle Marche, Dept. of Industrial Engineering and Mathematical Sciences, Ancona, Italy: Institute of Electrical and Electronics Engineers Inc.; 2023 2023. 6-10 p. ISBN: 9781665456937 (ISBN).

2.	Monteiro R, Rabello GCM, Moreno CR, Moitinho MS, Pires FA, Samesina N, et al. Enhancing cardiac postoperative care: a smartwatch-integrated remote telemonitoring platform for health screening with ECG analysis. Frontiers in Cardiovascular Medicine. 2024 2024;11. doi: 10.3389/fcvm.2024.1443998.

3.	Arslan B, Sener K, Guven R, Kapci M, Korkut S, Sutasir MN, et al. Accuracy of the Apple Watch in measuring oxygen saturation: comparison with pulse oximetry and ABG. Irish Journal of Medical Science. 2024 2024;193(1):477-83. doi: 10.1007/s11845-023-03456-w.

4.	Littell L, Roelle L, Dalal A, Van Hare GF, Orr WB, Miller N, et al. Assessment of Apple Watch Series 6 pulse oximetry and electrocardiograms in a pediatric population. PLOS Digit Health. 2022 2022-8;1(8):e0000051. doi: 10.1371/journal.pdig.0000051.

5.	Pätz C, Michaelis A, Markel F, Löffelbein F, Dähnert I, Gebauer RA, et al. Accuracy of the Apple Watch Oxygen Saturation Measurement in Adults and Children with Congenital Heart Disease. Pediatric Cardiology. 2023 2023;44(2):333-43. doi: 10.1007/s00246-022-02987-w.

6.	Rafl J, Bachman TE, Rafl-Huttova V, Walzel S, Rozanek M. Commercial smartwatch with pulse oximeter detects short-time hypoxemia as well as standard medical-grade device: Validation study. Digital Health. 2022 2022;8. doi: 10.1177/20552076221132127.

7.	Spaccarotella C, Polimeni A, Mancuso C, Pelaia G, Esposito G, Indolfi C. Assessment of Non-Invasive Measurements of Oxygen Saturation and Heart Rate with an Apple Smartwatch: Comparison with a Standard Pulse Oximeter. Journal of Clinical Medicine. 2022 2022;11(6). doi: 10.3390/jcm11061467.

8.	Turki AF, Section II, Universitas Islam I, Universitas Islam I, Chapter ICSI. An Investigation into the Accuracy of Blood Oxygen Saturation Measurement using Smart Watch Devices Among Pediatric Users in Saudi Arabia. King Abdul Aziz University, Center of Excellence in Intelligent Engineering Systems, Electrical and Computer Engineering Department, Jeddah, Saudi Arabia: Institute of Electrical and Electronics Engineers Inc.; 2023 2023. 29-33 p. ISBN: 9798350302424 (ISBN).

9.	Pipek LZ, Nascimento RFV, Acencio MMP, Teixeira LR. Comparison of SpO2 and heart rate values on Apple Watch and conventional commercial oximeters devices in patients with lung disease. Scientific Reports. 2021 2021;11(1). doi: 10.1038/s41598-021-98453-3.

10.	Helmer P, Hottenrott S, Wienböker K, Pryss R, Drosos V, Seitz AK, et al. Reliability of continuous vital sign monitoring in post-operative patients employing consumer-grade fitness trackers: A randomised pilot trial. Digital Health. 2024 2024;10. doi: 10.1177/20552076241254026.

11.	Jiang Y, Spies C, Magin J, Bhosai SJ, Snyder L, Dunn J. Investigating the accuracy of blood oxygen saturation measurements in common consumer smartwatches. PLOS Digit Health. 2023 2023-7;2(7):e0000296. doi: 10.1371/journal.pdig.0000296.

12.	Støve MP, Graversen AH, Sørensen J. Assessment of Noninvasive Oxygen Saturation in Patients With COPD During Pulmonary Rehabilitation: Smartwatch versus Pulse Oximeter. Respiratory Care. 2023 2023;68(8):1041-8. doi: doi:10.4187/respcare.10760.

13.	Rajakariar K, Buntine P, Ghaly A, Zhu ZC, Abeygunawardana V, Visakhamoorthy S, et al. Accuracy of Smartwatch Pulse Oximetry Measurements in Hospitalized Patients With Coronavirus Disease 2019. Mayo Clinic Proceedings: Digital Health. 2024 2024;2(1):152-8. doi: 10.1016/j.mcpdig.2024.02.001.

14.	Weis A, Leroy M, Jux C, Rupp S, Backhoff D. Oxygen saturation measurement in cyanotic heart disease with the Apple watch. Cardiology in the Young. 2024 2024. doi: 10.1017/S1047951124025216.

15.	Walzel S, Mikus R, Rafl-Huttova V, Rozanek M, Bachman TE, Rafl J. Evaluation of Leading Smartwatches for the Detection of Hypoxemia: Comparison to Reference Oximeter. Sensors (Basel). 2023 2023-11-14;23(22). doi: 10.3390/s23229164.

16.	Schiefer LM, Treff G, Treff F, Schmidt P, Schäfer L, Niebauer J, et al. Validity of peripheral oxygen saturation measurements with the garmin fēnix® 5x plus wearable device at 4559 m. Sensors. 2021 2021;21(19). doi: 10.3390/s21196363.

17.	Browne SH, Vaida F, Umlauf A, Kim J, DeYoung P, Owens RL. Performance of a commercial smart watch compared to polysomnography reference for overnight continuous oximetry measurement and sleep apnea evaluation. J Clin Sleep Med. 2024 2024-4-23. doi: 10.5664/jcsm.11178.

18.	Budig M, Stoohs R, Keiner M. Validity of Two Consumer Multisport Activity Tracker and One Accelerometer against Polysomnography for Measuring Sleep Parameters and Vital Data in a Laboratory Setting in Sleep Patients. Sensors. 2022 2022;22(23). doi: 10.3390/s22239540.

19.	Jung H, Kim D, Choi J, Joo EY. Validating a Consumer Smartwatch for Nocturnal Respiratory Rate Measurements in Sleep Monitoring. Sensors. 2023 2023;23(18). doi: 10.3390/s23187976.

20.	Jung H, Kim D, Lee W, Seo H, Seo J, Choi J, et al. Performance evaluation of a wrist-worn reflectance pulse oximeter during sleep. Sleep Health. 2022 2022;8(5):420-8. doi: 10.1016/j.sleh.2022.04.003.

21.	Hermand E, Coll C, Richalet JP, Lhuissier FJ. Accuracy and Reliability of Pulse O 2Saturation Measured by a Wrist-worn Oximeter. International Journal of Sports Medicine. 2021 2021;42(14):1268-73. doi: 10.1055/a-1337-2790.

22.	Khushhal AA, Mohamed AA, Elsayed ME. Accuracy of Apple Watch to Measure Cardiovascular Indices in Patients with Chronic Diseases: A Cross Sectional Study. Journal of Multidisciplinary Healthcare. 2024 2024;17:1053-63. doi: 10.2147/JMDH.S449071.

23.	Cao R, Azimi I, Sarhaddi F, Niela-Vilen H, Axelin A, Liljeberg P, et al. Accuracy Assessment of Oura Ring Nocturnal Heart Rate and Heart Rate Variability in Comparison With Electrocardiography in Time and Frequency Domains: Comprehensive Analysis. Journal of Medical Internet Research. 2022 2022;24(1). doi: 10.2196/27487.

24.	Kinnunen H, Rantanen A, Kentt T, Koskim ki H. Feasible assessment of recovery and cardiovascular health: Accuracy of nocturnal HR and HRV assessed via ring PPG in comparison to medical grade ECG. Physiological Measurement. 2020 2020;41(4). doi: 10.1088/1361-6579/ab840a.

25.	Miller DJ, Sargent C, Roach GD. A Validation of Six Wearable Devices for Estimating Sleep, Heart Rate and Heart Rate Variability in Healthy Adults. Sensors. 2022 2022;22(16). doi: 10.3390/s22166317.

26.	Bonneval L, Wing D, Sharp S, Tristao Parra M, Moran R, LaCroix A, et al. Validity of Heart Rate Variability Measured with Apple Watch Series 6 Compared to Laboratory Measures. Sensors. 2025 2025;25(8). doi: 10.3390/s25082380.

27.	Hernando D, Roca S, Sancho J, Alesanco Á, Bailón R. Validation of the apple watch for heart rate variability measurements during relax and mental stress in healthy subjects. Sensors (Switzerland). 2018 2018;18(8). doi: 10.3390/s18082619.

28.	Nuuttila OP, Korhonen E, Laukkanen J, Kyröläinen H. Validity of the Wrist-Worn Polar Vantage V2 to Measure Heart Rate and Heart Rate Variability at Rest. Sensors (Basel). 2021 2021-12-26;22(1). doi: 10.3390/s22010137.

29.	Rodrigues E, Lima D, Barbosa P, Gonzaga K, Guerra RO, Pimentel M, et al. HRV Monitoring Using Commercial Wearable Devices as a Health Indicator for Older Persons during the Pandemic. Sensors [Internet]. 2022; 22(5).

30.	Hermans F, Arents E, Blondeel A, Janssens W, Cardinaels N, Calders P, et al. Validity of a Consumer-Based Wearable to Measure Clinical Parameters in Patients With Chronic Obstructive Pulmonary Disease and Healthy Controls: Observational Study. JMIR mHealth and uHealth. 2024 2024;12. doi: 10.2196/56027.

31.	O'Grady B, Lambe R, Baldwin M, Acheson T, Doherty C. The Validity of Apple Watch Series 9 and Ultra 2 for Serial Measurements of Heart Rate Variability and Resting Heart Rate. Sensors (Basel). 2024 2024-9-26;24(19). doi: 10.3390/s24196220.

32.	Sarhaddi F, Kazemi K, Azimi I, Cao R, Niela-Vilén H, Axelin A, et al. A comprehensive accuracy assessment of Samsung smartwatch heart rate and heart rate variability. PLOS ONE. 2022;17(12):e0268361. doi: 10.1371/journal.pone.0268361.

33.	Falter M, Scherrenberg M, Driesen K, Pieters Z, Kaihara T, Xu L, et al. Smartwatch-Based Blood Pressure Measurement Demonstrates Insufficient Accuracy. Frontiers in Cardiovascular Medicine. 2022 2022;9. doi: 10.3389/fcvm.2022.958212.

34.	Poli A, Cosoli G, Iadarola G, Spinsante S, Scalise L, Instrumentation I, et al. Feasibility of Blood Pressure Measurement through Wearable Devices: Analysis of Smartwatches Performance. Polytechnic University of Marche, Dept. of Information Eng., Ancona, Italy Polytechnic University of Marche, Dept. of Industrial Eng. and Mathematical Sciences, Ancona, Italy: Institute of Electrical and Electronics Engineers Inc.; 2022 2022. ISBN: 9781665482998 (ISBN).

35.	Hahnen C, Freeman CG, Haldar N, Hamati JN, Bard DM, Murali V, et al. Accuracy of Vital Signs Measurements by a Smartwatch and a Portable Health Device: Validation Study. JMIR Mhealth Uhealth. 2020 2020/2/12;8(2):e16811. doi: 10.2196/16811.

36.	Lins L, do Nascimento EGC, da Silva Júnior JA, de Medeiros Fernandes TAA, de Andrade MF, de Mesquita Andrade C. Accuracy of wearable electronic device compared to manual and automatic methods of blood pressure determination. Med Biol Eng Comput. 2023 2023-10;61(10):2627-36. doi: 10.1007/s11517-023-02869-0.

37.	Yi L, Lv ZH, Hu SY, Liu YQ, Yan JB, Zhang H, et al. Validating the accuracy of a multifunctional smartwatch sphygmomanometer to monitor blood pressure. Journal of Geriatric Cardiology. 2022 2022;19(11):843-52. doi: 10.11909/j.issn.1671-5411.2022.11.004.

38.	Cosoli G, Antognoli L, Scalise L, Instrumentation I, Measurement S, Chapter ISCI, et al. Indirect Estimation of Breathing Rate through Wearable Devices. Marche Polytechnic University, Dept. of Industrial Eng. & Mathematical Sciences, Ancona, Italy: Institute of Electrical and Electronics Engineers Inc.; 2022 2022. ISBN: 9781665482998 (ISBN).
