# Supplementary material for: The Representation of Different Populations in Studies Assessing the Validity of Consumer Wearable Photoplethysmography-Based Measurements: Scoping Review
Source: JMIR Mhealth Uhealth. 2026 Aug 28;14:e87324. doi: 10.2196/87324 (PMC13524368; doi:10.2196/87324)
Supplement: Supplementary file 4 [file mhealth-v14-e87324-s003.docx]

## Multimedia Appendix 3: Results per source. Sources are presented in alphabetical order

| Source | First Author | Year | PPG-based measurements | Wearables | Physical activity | N | Sex (%f) | Age (years) | BMI (kg/m^2^) | Weight (kg) | Height (cm) | Skin tone (Fitzpatrick scale) |
| --- | --- | --- | --- | --- | --- | --- | --- | --- | --- | --- | --- | --- |
| [1] | Abdalazim | 2023 | PR | Oura Health Oy Oura ring | sleep | 5 | 60 | 26.2 ± 2.3 | NA | NA | NA | NA |
| [2] | AbiMansour | 2024 | PR | Apple Watch S7 | rest | 290 | 59.7 | NA | NA | NA | NA | NA |
| [3] | Abt | 2017 | PR | Apple Watch | vigorous | 15 | 46.7 | 32 ± 10 | NA | 73.5 ± 14.8 | 175 ± 8 | NA |
| [4] | Ahn | 2020 | PR | Samsung Gear S2 , LG Watch Urbane , Apple Watch | light | 4 | 25 | 24 | NA | NA | NA | NA |
| [5] | Alfonso | 2022 | PR | Apple Watch S6 , Polar Vantage M2 | rest , moderate | 18 | 44.4 | 22 ± 2.45 | NA | 65 ± 9.99 | 172.21 ± 8.95 | NA |
| [6] | Al-Kaisey | 2019 | PR | Fitbit Charge HR , Apple Watch S3 | mixed | 32 | 37.5 | 68 ± 12 | NA | NA | NA | NA |
| [7] | Antognoli | 2023 | PR, BP | Samsung Galaxy Watch 3 | rest , moderate , vigorous | 20 | 45 | 25.6 ± 3.6 | NA | NA | NA | NA |
| [8] | Arslan | 2023 | SpO_2_ | Apple Watch S6 | rest | 167 | 40.7 | 70.96 ± 11.51 | NA | 79.25 ± 20.33 | 166.29 ± 9.75 | NA |
| [9] | Baek | 2021 | PR | Fitbit Charge 2 | moderate , vigorous | 15 | 0 | 23.7 ± 3 | NA | NA | NA | NA |
| [10] | Bai | 2018 | PR | Apple Watch , Fitbit Charge HR | light , moderate | 39 | 41 | 32 ± 11 | 24.7 ± 4 | 72.7 ± 12.8 | 171.7 ± 9.5 | NA |
| [11] | Bai | 2021 | PR | Apple Watch S2 , Fitbit Alta , Fitbit Charge 2 | mixed | 48 | 64.6 | 26.8 ± 11.9 | 23.2 ± 3 | 66.3 ± 13 | 168.4 ± 10.4 | NA |
| [12] | Barrios | 2019 | PR, HRV | Fitbit Charge HR , Polar OH1+ , Whoo Tickr Fit | moderate , vigorous | 14 | 50 | NA | NA | 67 | 170 | NA |
| [13] | Benedetti | 2021 | PR | Fitbit Charge HR | sleep , mixed | 25 | 68 | 22.36 ± 3 | NA | NA | NA | NA |
| [14] | Benedetto | 2018 | PR | Fitbit Charge 2 | light , moderate , vigorous | 15 | 53.3 | 31.5 ± 4 | 23 ± 1.4 | 74.4 ± 9.7 | 174.8 ± 9 | NA |
| [15] | Bent | 2020 | PR, HRV | Apple Watch S4 , Fitbit Charge 2 , Garmin Vivosmart 3 , Xiaomi Mi Band 3 | rest , light , moderate | 53 | 64.2 | 25.6 | NA | NA | NA | NA |
| [16] | Bonneval | 2025 | HRV | Apple Watch | rest , light | 78 | 56.4 | 43.6 ± 18.8 | 24.7 ± 4.02 | 72.5 ± 14.8 | 171 ± 9.38 | NA |
| [17] | Boudreaux | 2018 | PR | Apple Watch S2 , Fitbit Blaze , Fitbit Charge 2 , Polar A360 , Garmin Vivosmart HR , TomTom Touch , Bose SoundSport Pulse | rest , moderate , vigorous | 50 | 56 | 22.4 ± 2.9 | 26.4 ± 4.3 | 76.9 ± 17.7 | 170.3 ± 10.6 | NA |
| [18] | Brazendale | 2019 | PR | Fitbit Charge HR | mixed | 19 | 47.4 | 8 ± 1.8 | NA | NA | NA | NA |
| [19] | Bretonneau | 2023 | PR | Polar Unite | rest , vigorous | 30 | 50 | 26.5 ± 9 | NA | 68.3 ± 12.2 | 171.5 ± 18.2 | NA |
| [20] | Bretonneau | 2024 |  |  |  |  |  |  |  |  |  |  |
| [21] | Browne | 2024 | SpO_2_ | Samsung Galaxy Watch 4 | sleep | 51 | 33.3 | NA | NA | NA | NA | NA |
| [22] | Budig | 2021 | PR | Garmin Forerunner 945 , Polar Ignite | rest , moderate , vigorous | 36 | 44.4 | 36.1 ± 12.8 | 23.4 ± 2.7 | 73.3 ± 14.4 | 176.1 ± 96 | NA |
| [23] | Budig | 2022 | PR, HRV, SpO_2_, RR | Garmin Forerunner 945 , Polar Ignite | sleep | 49 | 38.8 | 55.01 ± 10.19 | 30.57 ± 6.32 | 94.71 ± 19.36 | 176.2 ± 10.24 | 2.51 ± 0.68 |
| [24] | Cadmus-Bertram | 2017 | PR | Fitbit Surge , Basis Peak , Fitbit Charge HR , Mio Fuse | rest , moderate | 40 | 50 | 49.3 ± 9.5 | 25.1 ± 3.9 | NA | NA | NA |
| [25] | Cao | 2022 | PR, HRV | Oura Health Oy Oura ring | Sleep | 46 | 50 | 32.3 ± 6.4 | 24.8 ± 4.5 | NA | NA | NA |
| [26] | Carrier | 2025 | SpO_2_ | Garmin Fenix 6 | rest | 22 | 40.9 | 25.48 ± 6.02 | 22.91 ± 2.4 | 68.88 ± 9.1 | 173.27 ± 7.7 | NA |
| [27] | Ceugniez | 2025 | PR | Fitbit Charge 4 | moderate , vigorous | 26 | 42.3 | 21.1 ± 5.8 | 21.9 ± 1.9 | 68.1 ± 12.9 | 176 ± 11 | 2.9 ± 0.6 |
| [28] | Chase | 2024 | PR | Apple Watch S9 | rest , light , moderate | 30 | 36.7 | 20.7 ± 1.1 | NA | 70.4 ± 11 | 169.1 ± 7.7 | NA |
| [29] | Choi | 2019 | PR | Fitbit Charge 2 | moderate | 13 | 100 | 55.92 ± 3.07 | 22.51 ± 2.43 | 57.76 ± 8.09 | NA | NA |
| [30] | Choksatchawathi | 2020 | PR | Fitbit Charge HR , Apple Watch S4 , Mobvoi TicWatch Pro 5 | rest , vigorous | 29 | 41.4 | 24.62 ± 3.92 | NA | 61.17 ± 13.96 | 162.25 ± 19.01 | NA |
| [31] | Chow | 2020 | PR | Xiaomi Mi Band 2 , Garmin Vivosmart HR+ | rest , moderate | 40 | 50 | NA | NA | NA | NA | NA |
| [32] | Claes | 2017 | PR | Garmin Forerunner 225 | rest , moderate | 12 | 50 | 28 ± 4.79 | 22.14 ± 3.46 | 69.28 ± 13.24 | 176.23 ± 10.97 | NA |
| [33] | Climstein | 2020 | PR | Polar Vantage M | rest , moderate , vigorous | 29 | 44.8 | 26.14 ± 3.43 | 24.18 ± 2.8 | 74.25 ± 9.7 | 175 ± 7 | NA |
| [34] | Coelli | 2024 | PR | Fitbit Sense | rest , light | 10 | 40 | NA | 21.4 ± 2.1 | NA | NA | NA |
| [35] | Collins | 2019 | PR | Garmin Vivosmart 3 | rest , light , moderate , mixed | 4 | 50 | 38.25 ± 12.58 | 23.43 ± 2.3 | 67.25 ± 6.6 | 169 ± 4.6 | NA |
| [36] | Cosoli | 2022 | RR | Garmin Venu Sq | rest | 4 | 100 | 24 ± 1.2 | 20.88 ± 0.62 | NA | NA | NA |
| [37] | Cosoli | 2023 | PR | Samsung Galaxy Watch 3 | rest , light , vigorous | 30 | 63.3 | 22 ± 3 | 22.5 ± 2.3 | 64 ± 9 | 169 ± 0.07 | NA |
| [38] | Cosoli | 2022 | PR | Garmin Venu Sq , Polar Vantage V2 | rest , moderate , vigorous | 10 | NA | 17 ± 3 | 19.8 ± 1.21 | 52.1 ± 4.72 | 162.1 ± 3.78 | NA |
| [39] | Danielsson | 2024 | PR | Apple Watch S4 , Fitbit Versa | rest , moderate , vigorous | 40 | 45 | 35.3 ± 11.8 | 24.5 ± 4.1 | 74.8 ± 15.2 | 174.5 ± 10.9 | NA |
| [40] | De Oliveira Damasceno | 2022 | PR | Garmin 735XT | rest , light , moderate , vigorous | 28 | 0 | 23.41 ± 3.43 | 23.9 ± 1.92 | 74.3 ± 8.67 | 176 ± 8 | NA |
| [41] | De Zambotti | 2016 | PR | Fitbit Charge HR | sleep | 32 | 46.9 | 17.3 ± 2.5 | 22.2 ± 3.6 | NA | NA | NA |
| [42] | Dondzila | 2018 | PR | Fitbit Charge HR , Mio Fuse | rest , moderate , vigorous | 40 | 57.5 | 21.6 ± 2 | 24.6 ± 4.2 | NA | NA | NA |
| [43] | Dooley | 2017 | PR | Apple Watch , Fitbit Charge HR , Garmin Forerunner 225 | rest , moderate , vigorous | 62 | 58.1 | 22.55 ± 4.34 | 24.6 ± 4.77 | 72.02 ± 18.99 | 170 ± 11 | NA |
| [44] | Düking | 2020 | PR | Apple Watch S4 , Polar Vantage V , Garmin Fenix 5 , Fitbit Versa | rest , moderate , vigorous | 25 | 60 | 26 ± 7 | NA | 70.1 ± 12 | 174 ± 10 | NA |
| [45] | El-Amrawy | 2015 | PR | Apple Watch , Motorola Moto 360 , Samsung Gear Fit , Samsung Gear 2 , Samsung Gear 3 | rest , moderate | 4 | 0 | 26.5 ± 12.8 | NA | NA | NA | NA |
| [46] | Etiwy | 2019 | PR | Fitbit Blaze , Apple Watch , Garmin Forerunner 235 , TomTom Spark Cardio | rest , moderate | 80 | 18.8 | 62 ± 13 | 29 ± 5.5 | 88 ± 19 | 174 ± 8.9 | NA |
| [47] | Falter | 2019 | PR | Apple Watch | rest , moderate , vigorous | 40 | 20 | 61.9 ± 15.2 | 27 ± 5 | 79 ± 16.2 | 171.1 ± 9.3 | NA |
| [48] | Falter | 2022 | BP | Samsung Galaxy Watch Active 2 | rest | 40 | 42.5 | 57.7 ± 12.5 | 28.2 ± 4.6 | NA | NA | NA |
| [49] | Ferrera | 2018 | PR | Mobvoi TicWatch S2 , Motorola Moto 360 , Huawei Watch | NA | 25 | 48 | NA | NA | NA | NA | NA |
| [50] | Flett | 2024 | PR | Garmin Venu SQ | rest , light , moderate , vigorous | 18 | 44.4 | NA | NA | NA | NA | NA |
| [51] | Gagnon | 2022 | PR | Fitbit Charge 2 | light | 34 | 61.8 | 26.8 ± 8.5 | NA | NA | NA | NA |
| [52] | Gaynor | 2019 | PR | Fitbit Charge HR | rest , moderate , vigorous | 15 | 26.7 | 34 ± 11.2 | 23.6 ± 3.2 | NA | NA | NA |
| [53] | Ge | 2016 | PR | Apple Watch | rest , moderate , vigorous | 50 | 30 | NA | NA | NA | NA | NA |
| [54] | Giggins | 2021 | PR | Apple Watch S5 | rest , moderate , vigorous | 10 | 50 | 30.4 ± 8 | NA | 74.7 ± 12.1 | 174 ± 9.4 | 2.8 ± 1.25 |
| [55] | Gilgen-Ammann | 2021 | PR | Garmin Fenix 3 | rest , moderate , vigorous | 23 | 0 | 20.9 ± 2.5 | 24.2 ± 2.5 | 76 ± 11.6 | 180 ± 10 | NA |
| [56] | Gillinov | 2017 | PR | Scosche Rhythm+ , Fitbit Blaze , Apple Watch , Garmin Forerunner 235 , TomTom Spark Cardio | rest , light , moderate , vigorous | 50 | 54 | 38 ± 12 | 25 ± 3.5 | 73 ± 16 | 171 ± 11 | NA |
| [57] | Gorny | 2017 | PR | Fitbit Charge HR | mixed | 10 | 30 | 25.4 ± 3.7 | 22.9 ± 3.8 | NA | NA | NA |
| [58] | Haghayegh | 2019 | PR | Fitbit Charge 2 | sleep | 35 | 48.6 | 35.2 ± 10.6 | 22.4 ± 3 | NA | NA | NA |
| [59] | Hahnen | 2020 | PR, BP | Everlast TR10 | rest | 85 | 42.4 | 53 ± 21 | 28 ± 7 | NA | NA | NA |
| [60] | Hänsel | 2018 | PR | Apple Watch S2 , Microsoft Band 2 | rest , light | 21 | 38.1 | 28.9 ± 4.5 | NA | NA | NA | NA |
| [61] | Haveman | 2022 | PR | Fitbit Charge 3 | rest , light , moderate | 20 | 45 | NA | NA | NA | NA | NA |
| [62] | Held | 2022 | PR | Apple Watch S4 , Fitbit Charge | light , moderate , vigorous | 16 | 81.2 | 32.8 ± 11.1 | 23.6 ± 2.9 | 66.5 ± 11.4 | 167.7 ± 9.7 | NA |
| [63] | Helmer | 2022 | PR | Apple Watch S7 , Fitbit Sense , Garmin Fenix 6 Pro , Withings ScanWatch | rest , sleep | 112 | 37.5 | NA | NA | NA | NA | NA |
| [64] | Helmer | 2024 | PR, SpO_2_, RR | Apple Watch S7 , Garmin Fenix 6 , Withings ScanWatch | rest , sleep | 26 | 46.2 | NA | NA | NA | NA | NA |
| [65] | Hermand | 2021 | PR, SpO_2_ | Garmin Forerunner 245 | rest , moderate | 10 | 50 | 32.5 ± 9.4 | NA | 72.5 ± 15.5 | 172.8 ± 10.3 | 1.9 ± 1.4 |
| [66] | Hermans | 2024 | PR, HRV, SpO_2_, RR | Fitbit Charge 4 | mixed | 55 | 21.8 | NA | NA | NA | NA | NA |
| [67] | Hernando | 2018 | HRV | Apple Watch | rest , light | 20 | NA | NA | NA | NA | NA | NA |
| [68] | Hettiarachchi | 2019 | PR | Polar OH1 | rest , moderate , vigorous | 24 | 50 | 28.09 ± 5.5 | 22.6 ± 5.33 | 66.5 ± 14.65 | 170.72 ± 9.98 | NA |
| [69] | Ho | 2022 | PR | Garmin Forerunner 945 , Apple Watch S6 | rest , moderate , vigorous | 30 | 50 | 29.3 ± 3.6 | 21.2 ± 2.3 | 60.1 ± 10.7 | 168 ± 7.9 | NA |
| [70] | Hoevenaars | 2022 | PR | Fitbit Charge 2 | rest , light , moderate , vigorous | 44 | 20.5 | 48.9 ± 12 | 24.2 ± 4.1 | NA | NA | NA |
| [71] | Horton | 2017 | PR | Polar M600 | rest , moderate , vigorous | 36 | 50 | 40.5 ± 9.6 | 23.3 ± 2.94 | 69.8 ± 11 | 172.6 ± 9.2 | 2.5 ± 0.86 |
| [72] | Hough | 2017 | PR | Apple Watch S1 , Fitbit Charge HR , TomTom Touch , Mio Fuse | light , moderate , vigorous | 18 | 11.1 | 30 ± 8 | 24.1 ± 2.8 | 75.3 ± 12.8 | 176 ± 8 | NA |
| [73] | Hung | 2025 | PR | Fitbit Charge 5 | rest , light , moderate , vigorous | 25 | 64 | 25.8 ± 1.9 | 23.7 ± 3.4 | 70.6 ± 13.6 | 172.3 ± 7.4 | NA |
| [74] | Huynh | 2021 | PR | Apple Watch | rest | 20 | 15 | 66 ± 6.5 | 33.2 ± 4.8 | NA | NA | NA |
| [75] | Hwang | 2019 | PR | Apple Watch S2 , Samsung Gear S3 , Fitbit Charge 2 | rest | 51 | 51 | 44.4 ± 16.6 | 23.7 ± 3 | 64.5 ± 9.1 | 163.1 ± 16.4 | NA |
| [76] | Ibrahim | 2024 | PR | AMAZFIT Cor , AMAZFIT Bip | rest , light , moderate , vigorous | 51 | 21.6 | 61 ± 12 | 26 ± 5 | NA | NA | 3 ± 1 |
| [77] | Icenhower | 2025 | PR | Garmin Forerunner 45 | rest , light , moderate | 29 | 48.3 | 22.24 ± 4.54 | 23.19 ± 3.6 | NA | NA | NA |
| [78] | Imai | 2025 | PR | Apple Watch S7 | rest , moderate | 15 | 0 | 30.1 ± 4.53 | 21.4 ± 2.1 | 65.3 ± 7.89 | 174.5 ± 6.76 | NA |
| [79] | Jachymek | 2021 | PR | Fitbit Charge 4 , Xiaomi Mi Band 5 | rest , moderate , vigorous | 31 | 32.3 | 28 | 23.5 | NA | 177.7 ± 14.62 | NA |
| [80] | Jaiswal | 2024 | PR, HRV | Samsung Galaxy Watch 4 | rest , light | 20 | NA | NA | NA | NA | NA | NA |
| [81] | Jamieson | 2024 | PR | Garmin Vivoactive 4 , Fitbit Sense | rest , moderate | 16 | 56.2 | NA | NA | NA | NA | 2 ± 2.4 |
| [82] | Jarchi | 2018 | SpO_2_ | Huawei Watch 2 | rest | 5 | NA | NA | NA | NA | NA | NA |
| [83] | Jiang | 2023 | SpO_2_ | Apple Watch S7 , Garmin Venu 2S , Garmin Fenix 6 Pro , Withings ScanWatch | rest | 49 | 36.7 | NA | NA | NA | NA | NA |
| [84] | Jo | 2016 | PR | Basis Peak , Fitbit Charge HR | rest , moderate , vigorous | 24 | 50 | 24.8 ± 2.1 | NA | 71.3 ± 9.9 | 166 ± 7 | NA |
| [85] | Jørgensen | 2023 | PR | Garmin Vivosmart 4 | moderate , vigorous | 5 | 0 | 31.6 ± 6 | 19 ± 4.3 | 47.4 ± 6.3 | 159.5 ± 12.6 | NA |
| [86] | Jung | 2023 | RR | Samsung Galaxy Watch 4 , Samsung Galaxy Watch 5 | sleep | 195 | 26.7 | 48.9 ± 13.3 | 25.9 ± 4 | NA | NA | NA |
| [87] | Jung | 2022 | SpO_2_ | Samsung Galaxy Watch 4 | sleep | 97 | 23.7 | 44.4 ± 13 | 26.3 ± 4.4 | NA | NA | NA |
| [88] | Jung | 2020 | PR | Fitbit Charge HR | rest , light , moderate , vigorous | 32 | 53.1 | 26.03 ± 6.59 | 26.4 ± 5.11 | 74.09 ± 15.89 | 167.38 ± 8.3 | NA |
| [89] | Kang | 2019 | PR | Fitbit Charge HR | rest , light , moderate , vigorous | 43 | 41.9 | 9.7 ± 1.3 | 17.9 ± 2.2 | 37.8 ± 8.1 | 144.5 ± 9.6 | NA |
| [90] | Khushhal | 2017 | PR | Apple Watch | rest , moderate , vigorous | 21 | 0 | 31.4 ± 7.2 | 26.1 ± 2.9 | NA | NA | NA |
| [91] | Khushhal | 2024 | PR, SpO_2_ | Apple Watch S8 | rest , moderate | 41 | 0 | 51 ± 7 | NA | NA | NA | NA |
| [92] | Kim | 2023 | PR | Apple Watch S7 , Samsung Galaxy Watch 4 | rest , moderate , vigorous | 44 | 18.2 | 60.9 ± 7.9 | 25.5 ± 2.9 | 70 ± 10.9 | 165.5 ± 8 | NA |
| [93] | Kinnunen | 2020 | PR, HRV | Oura Health Oy Oura ring | sleep | 60 | 66.7 | 31.6 ± 11.8 | NA | NA | NA | NA |
| [94] | Kondama Reddy | 2018 | PR | Garmin Vivosmart HR+ , Fitbit Charge 2 | rest , moderate , vigorous | 20 | 55 | 27.5 ± 6 | 22.5 ± 2.3 | 67.9 ± 10.8 | 173.2 ± 9.5 | NA |
| [95] | Koshy | 2018 | PR | Apple Watch S1 , Fitbit Blaze | rest | 102 | 35.3 | 68 ± 15 | NA | NA | NA | NA |
| [96] | Kozak | 2017 | PR | Fitbit Charge 2 | mixed | 15 | 40 | 75.35 | NA | NA | NA | NA |
| [97] | Kroll | 2016 | PR | Fitbit Charge HR | rest | 50 | 48 | 64 | NA | NA | NA | NA |
| [98] | Lauterbach | 2020 | PR, SpO_2_ | Garmin Fenix 5X | rest | 23 | 56.5 | 20.4 ± 2.1 | NA | 71.8 ± 11.1 | 170.2 ± 8 | NA |
| [99] | Lee | 2022 | PR | Apple Watch S4 | rest | 200 | 35.5 | 65.6 ± 14.6 | NA | NA | NA | NA |
| [100] | Leth | 2017 | PR | Fitbit Charge HR | sleep | 9 | 55.6 | 26 ± 5.1 | NA | NA | NA | NA |
| [101] | Lima | 2024 | PR | Samsung Galaxy Watch 4 | rest , light , moderate , vigorous | 14 | 50 | 28.6 ± 5.6 | NA | 70.6 ± 13.6 | 169 ± 6 | NA |
| [102] | Lins | 2023 | BP | Samsung Galaxy Watch 4 | rest | 22 | 54.5 | 21.1 ± 0.35 | NA | NA | NA | NA |
| [103] | Littell | 2022 | PR, SpO_2_ | Apple Watch S6 | rest | 84 | 54.8 | 7.2 | NA | 31.5 | 114.8 | NA |
| [104] | Martín-Escudero | 2023 | PR | TomTom Runner Cardio , Apple Watch , Samsung Gear S2 , Fitbit Charge HR | rest , moderate , vigorous | 8 | 12.5 | 35 ± 9.53 | 22.54 ± 2.51 | 67.53 ± 10.55 | 173 ± 7 | NA |
| [105] | Marzano-Felisatti | 2024 | PR | Coospo HW807 | light , moderate , vigorous | 30 | 0 | 20 ± 0 | NA | 77.8 ± 11.2 | 177.2 ± 5 | NA |
| [106] | Merrigan | 2023 | PR | Garmin Fenix 6 Pro , Polar Grit X Pro , Polar Verity Sense | rest , moderate , vigorous | 8 | 12.5 | 27.2 ± 7.4 | 24.5 ± 1.6 | 71.4 ± 4 | 171.4 ± 8 | NA |
| [107] | Meza | 2023 | PR | Fitbit Charge 5 | rest | 70 | 62.9 | 79.4 ± 10.1 | 26.9 ± 5.8 | NA | NA | NA |
| [108] | Miller | 2022 | PR, HRV | Apple Watch S6 , Garmin Forerunner 245 Music , Polar Vantage V , Oura Health Oy Oura ring , WHOOP WHOOP 3.0 | sleep | 53 | 49.1 | 25.4 ± 5.9 | NA | NA | NA | NA |
| [109] | Mink | 2025 | PR | Garmin Vivosmart 4 | rest | 26 | 73.1 | 25.23 | NA | NA | NA | NA |
| [110] | Moayedi | 2017 | PR | Apple Watch , Fitbit NA | moderate , vigorous | 10 | NA | NA | NA | NA | NA | NA |
| [111] | Montalvo | 2023 | PR | Apple Watch S6 , Apple Watch S7 | rest , light , moderate , vigorous | 44 | 22.7 | 23.03 ± 2.3 | 25.7 ± 5.6 | 81.5 ± 17.6 | 177 ± 9 | NA |
| [112] | Monteiro | 2024 | PR, BP, SpO_2_ | Samsung Galaxy Watch 5 | rest | 55 | 40 | 52.71 ± 9.92 | NA | NA | NA | NA |
| [113] | Montes | 2019 | PR | Samsung Gear 2 , Fitbit Surge , Garmin Vivosmart HR+ , Polar A360 , Scosche Rhythm+ | rest , moderate , vigorous | 40 | 50 | 25.09 ± 7.17 | 26.43 ± 5.19 | 77.19 ± 19.2 | 169.64 ± 11.18 | NA |
| [114] | Montoye | 2017 | PR | Fitbit Charge HR | rest , light , moderate , vigorous | 32 | 43.8 | 23.5 ± 7.35 | 25.6 ± 4.53 | 79.3 ± 2.05 | 175.8 ± 8.62 | NA |
| [115] | Muggeridge | 2021 | PR | Polar OH1 , Fitbit Charge 3 | rest , light , moderate , vigorous | 20 | 45 | 40 ± 10 | NA | 71.6 ± 11 | 173 ± 10 | NA |
| [116] | Müller | 2019 | PR | Polar A370 , Tempo Tempo HR | rest , moderate , vigorous , mixed | 55 | 47.3 | 30.5 ± 9.8 | NA | NA | NA | NA |
| [117] | Nash | 2024 | PR | Apple Watch S5 , Apple Watch S6 | rest | 80 | 50 | 13 | NA | 52 | 150 | NA |
| [118] | Navalta | 2023 | PR | Polar OH1 , Polar Verity Sense , Garmin Instrinct , Polar Vantage M2 | rest , moderate , vigorous | 18 | 55.6 | 26 ± 8 | 25.6 ± 3 | 73.3 ± 11.9 | 166.4 ± 3.5 | NA |
| [119] | Navalta | 2020 | PR | Garmin Fenix 5 , Jabra Elite Sport , Motiv Motiv Ring | vigorous | 21 | 47.6 | 31 ± 11 | NA | 75.6 ± 12.9 | 173 ± 6.9 | NA |
| [120] | Navalta | 2024 | PR | Garmin Instict , Polar Vantage M2 , Polar OH1 , Polar Verity sense | vigorous | 20 | 35 | 44.45 ± 19.6 | NA | 71.35 ± 11.4 | 173 ± 8.8 | NA |
| [121] | Neudorfer | 2024 | PR | Garmin Venu 2S , Polar Verity Sense , Polar Vantage M2 , Scosche Rhythm24 | rest , light , moderate , vigorous , mixed | 32 | 34.4 | 59.1 ± 10.1 | 26.1 ± 4.4 | 79 ± 16.5 | 173.3 ± 7.8 | NA |
| [122] | Nissen | 2022 | PR | Fitbit Charge 4 , Samsung Galaxy Watch Active 2 | rest , light , moderate , vigorous | 23 | 43.5 | 24.2 ± 4.6 | NA | 71 ± 12.75 | 175.7 ± 10.48 | NA |
| [123] | Nuss | 2019 | PR | Apple Watch S1 | rest , moderate , vigorous | 30 | 50 | 22.47 ± 3.69 | 22.43 ± 1.87 | NA | NA | NA |
| [124] | Nuuttila | 2021 | PR, HRV | Polar Vantage V2 | rest , sleep | 41 | 48.8 | 36 ± 7 | NA | NA | NA | NA |
| [125] | O’Driscoll | 2020 | PR | Fitbit Charge 2 | rest , moderate , vigorous | 59 | 69.5 | 44.42 ± 14.14 | NA | 75.65 ± 13.63 | 167.45 ± 8.91 | NA |
| [126] | O'Grady | 2024 | PR, HRV | Apple Watch S9 , Apple Watch Ultra 2 | rest | 39 | 56.4 | 24.6 ± 8.2 | 23.5 ± 1.97 | 73.08 ± 10.52 | 169 ± 34 | NA |
| [127] | Page | 2024 | PR | Fitbit Inspire HR | rest , light , moderate , vigorous | 14 | 100 | 63.07 ± 8.66 | 29.14 ± 4.71 | NA | NA | NA |
| [128] | Pasadyn | 2019 | PR | Apple Watch S3 , Fitbit Iconic , Garmin Vivosmart HR , TomTom Spark 3 | rest , moderate , vigorous | 50 | 32 | 29.5 ± 9.32 | 22.8 ± 2.4 | 69.4 ± 10.66 | 174.2 ± 9 | NA |
| [129] | Paslı | 2024 | PR | Apple Watch S7 | rest | 721 | 46 | 66 ± 17.1 | NA | NA | NA | NA |
| [130] | Passler | 2019 | PR | Bragi Dash Pro , Cosinuss consinnusOne | rest , moderate , vigorous | 20 | 30 | 22.3 ± 2 | NA | 69.6 ± 11 | NA | NA |
| [131] | Pätz | 2023 | SpO_2_ | Apple Watch S6 | rest | 508 | NA | NA | NA | NA | NA | NA |
| [132] | Pelizzo | 2018 | PR | Fitbit Charge HR | sleep | 30 | 46.7 | 8.21 ± 3.09 | 20.5 ± 5 | 31.3 ± 15.3 | 132.2 ± 23.3 | NA |
| [133] | Pierick | 2025 | PR | Garmin Vivosmart 4 , Fitbit Charge 4 | rest , moderate , vigorous | 47 | 44.7 | NA | NA | NA | NA | NA |
| [134] | Pipek | 2021 | PR, SpO_2_ | Apple Watch S6 | rest | 100 | 66 | 57.6 | NA | NA | NA | NA |
| [135] | Poli | 2022 | BP | Samsung Galaxy Watch 3 , Asus VivoWatch SP | rest | 19 | 47.4 | NA | NA | 70.84 ± 13.75 | 173.74 ± 10 | NA |
| [136] | Pope | 2019 | PR | Apple Watch , Fitbit Surge , TomTom Multisport Cardio Watch , Microsoft Band | moderate , vigorous | 21 | 66.7 | 24.5 ± 3.5 | NA | 64.4 ± 8.9 | 171 ± 8.3 | NA |
| [137] | Powierza | 2017 | PR | Fitbit Charge HR | rest , moderate , vigorous | 22 | 54.5 | 22 ± 2 | NA | 71.16 ± 17.34 | 175.09 ± 14.73 | NA |
| [138] | Rafl | 2022 | SpO_2_ | Apple Watch S6 | rest | 24 | 20.8 | 24 ± 2 | NA | 77 ± 11 | 181 ± 8 | NA |
| [139] | Rajakariar | 2024 | SpO_2_ | Apple Watch S7 , Withings ScanWatch | rest | 200 | 46 | 66 ± 18 | NA | NA | NA | NA |
| [140] | Ravanelli | 2025 | PR | Fitbit Charge 5 | mixed | 26 | 53.8 | 30.1 ± 14.2 | 24.9 ± 2.8 | 75.9 ± 11 | 174 ± 9 | 2.8 ± 0.9 |
| [141] | Ray | 2021 | PR | Motorola Moto 360 , LG Watch Urbane , Polar M600 , Huawei Watch 2 , Mobvoi TicWatch Pro , Fossil Carlyle HR , Misfit Vapor X | rest | 18 | 66.7 | 26.5 | NA | NA | NA | NA |
| [142] | Reece | 2021 | PR | Jabra Elite , Scosche Rhythm 24 , Apple Watch S4 , Garmin Forerunner 735 XT | rest , light , moderate , vigorous | 23 | 43.5 | 23.57 ± 3.22 | 24.87 ± 4.45 | 73.4 ± 16.17 | 171.53 ± 10.52 | NA |
| [143] | Rho | 2024 | PR, HRV | Samsung Galaxy Watch 5 | rest , light | 20 | 35 | 39 ± 15 | NA | NA | NA | NA |
| [144] | Rodrigues | 2022 | HRV | Fitbit Inspire | rest | 10 | NA | NA | NA | NA | NA | NA |
| [145] | Rozanski | 2018 | PR | Fitbit Charge HR , Garmin Vivosmart | mixed | 37 | 35.1 | 64.4 ± 15 | NA | 77.1 ± 15.4 | 171 ± 9.3 | NA |
| [146] | Ruiz-Malagón | 2023 | PR | Polar Ignite | rest , moderate , vigorous | 40 | 20 | 40 ± 15.68 | 24.12 ± 3.83 | 73.43 ± 15.68 | 173.93 ± 11.12 | NA |
| [147] | Ruiz-Malagón | 2021 | PR | Polar Vantage M | vigorous | 37 | 40.5 | 22 ± 9 | 19.66 ± 2.63 | 56.2 ± 12.8 | 167.6 ± 11.8 | NA |
| [148] | Saghir | 2020 | PR | Apple Watch S4 | rest | 43 | 65.1 | 31 ± 8.46 | NA | NA | NA | NA |
| [149] | Sañudo | 2019 | PR | Apple Watch | rest , moderate , vigorous | 45 | 0 | 24 ± 4 | NA | 72.2 ± 5.8 | 177 ± 5 | NA |
| [150] | Sarhaddi | 2022 | PR, HRV | Samsung Gear Sport | sleep , mixed | 28 | 50 | 32.5 ± 6.6 | 25.4 ± 5.2 | NA | NA | NA |
| [151] | Schiefer | 2021 | SpO_2_ | Garmin Fenix 5X Plus | rest , moderate , vigorous | 13 | 15.4 | 57 ± 6 | 24.8 ± 3.3 | 76 ± 11 | 175 ± 7 | NA |
| [152] | Schweizer | 2025 | PR | Polar Verity sense , Polar Vantage V2 | rest , light , moderate , vigorous | 16 | 43.8 | 27.4 ± 5.8 | 23.1 ± 2 | 69.9 ± 9.4 | 173.5 ± 9.2 | NA |
| [153] | Sequeira | 2020 | PR | Apple Watch , Fitbit Charge HR , Garmin Vivosmart HR , Polar A360 | rest | 65 | 53.8 | 52.3 ± 17.2 | NA | NA | NA | NA |
| [154] | Seshadri | 2020 | HR_w_ | Apple Watch S4 | rest | 50 | 28 | 61.4 ± 10.4 | 30.4 ± 4.87 | NA | NA | NA |
| [155] | Shcherbina | 2017 | PR | Apple Watch , Basis Peak , Samsung Gear S2 , Fitbit Surge , Microsoft Band , Mio Alpha 2 | rest , moderate , vigorous | 60 | 51.7 | 38 ± 10.64 | 23.6 ± 3.1 | 70.6 ± 15.9 | 172.2 ± 10.2 | 3.7 ± 1.31 |
| [156] | Shumate | 2021 | PR | Polar Vantage M | rest , moderate , vigorous | 30 | 43.3 | 26.1 ± 3.39 | NA | 73.59 ± 11.9 | 52.36 ± 7.4 | NA |
| [157] | Sjoberg | 2021 | PR | Fitbit Versa | rest , light , moderate , mixed | 42 | 76.2 | 43.8 ± 11.8 | 29.4 ± 5.8 | NA | NA | NA |
| [158] | Spaccarotella | 2022 | SpO_2_ | Apple Watch S6 | rest | 257 | 34.6 | 64 ± 16.2 | 27.1 ± 4.7 | 75 ± 15.3 | 166.3 ± 8.4 | NA |
| [159] | Spierer | 2015 | PR | Omron HR500U , Mio Alpha | rest , light , moderate , vigorous | 47 | 42.6 | 28.4 ± 10.1 | NA | 73.4 ± 14.2 | 172 ± 9.4 | 3 ± 0.7 |
| [160] | Spinsante | 2019 | PR | Samsung Gear Fit | rest , light | 4 | 50 | 37.8 ± 14.4 | NA | 65.8 ± 7.69 | 167 ± 25 | NA |
| [161] | Stahl | 2016 | PR | Scosche Rhythm , Mio Alpha , Fitbit Charge HR , Basis Peak , Microsoft Band , TomTom Runner Cardio | rest , moderate , vigorous | 50 | 36 | 26.3 ± 5.9 | 24.69 ± 3.42 | 75.64 ± 13.7 | 174.52 ± 10 | NA |
| [162] | Stone | 2021 | PR, HRV | Oura Health Oy Oura ring gen 2 | rest | 5 | 40 | 20 ± 1.6 | NA | 68.9 ± 13 | NA | NA |
| [163] | Støve | 2023 | SpO_2_ | Apple Watch S7 , Garmin Vivosmart 4 | rest | 36 | 55.6 | 72.3 ± 7.9 | 28 ± 6.7 | 81.1 ± 23.6 | 170 ± 9.6 | NA |
| [164] | Støve | 2022 | PR | Apple Watch S6 , WHOOP WHOOP band | vigorous | 29 | 55.2 | 24.5 ± 4 | 21.1 ± 2.8 | 74.8 ± 12.7 | 177 ± 10 | NA |
| [165] | Støve | 2019 | PR | Garmin Forerunner 235 | rest , moderate , vigorous | 29 | 41.4 | 29 ± 9.4 | 23.7 ± 2.2 | 73 ± 10.5 | 175 ± 9 | NA |
| [166] | Støve | 2020 | PR | Apple Watch S2 , Garmin Forerunner 235 | rest , moderate , vigorous | 30 | 50 | 24.8 ± 6.3 | 23.1 ± 1.95 | 70.5 ± 9.4 | 174 ± 8 | NA |
| [167] | Stucky | 2021 | PR | Fitbit Charge 2 | sleep | 59 | 55.9 | 33.5 ± 8.1 | 23.9 ± 2.9 | NA | NA | NA |
| [168] | Tedesco | 2019 | PR | Fitbit Charge 2 , Garmin Vivosmart HR+ , Philips Health Watch , Withings Pulse Ox | rest , light , moderate | 18 | 61.1 | 69.4 ± 2.7 | NA | 71.4 ± 8 | 167.9 ± 8.1 | NA |
| [169] | Theurl | 2023 | HRV | Garmin Vivoactive 4 | rest | 263 | 27 | NA | NA | NA | NA | NA |
| [170] | Thiebaud | 2018 | PR | Fitbit Surge , Microsoft Band , TomTom Cardio | moderate , vigorous | 22 | 9.1 | 22 ± 3 | NA | 75.9 ± 10.2 | 1.72 ± 9 | NA |
| [171] | Thomson | 2019 | PR | Fitbit Charge HR , Apple Watch | rest , light , moderate , vigorous | 30 | 50 | 23.5 ± 2.96 | 22.84 ± 2.23 | NA | NA | NA |
| [172] | Turki | 2021 | HRV | Apple Watch S3 | rest | 6 | 33.3 | 26.4 ± 3.2 | NA | NA | NA | NA |
| [173] | Turki | 2023 | SpO_2_ | Apple Watch S6 , Samsung Galaxy Watch 4 | rest | 100 | 43 | 13 ± 3.3 | 21.07 ± 6.3 | NA | NA | 3 ± 0.91 |
| [174] | Turki | 2019 | PR | Apple Watch S3 , Samsung Gear S3 , Fitbit Charge 2 , Polar M600 | rest , moderate | 5 | 40 | 26.4 ± 3.2 | 24.7 ± 1.92 | NA | NA | NA |
| [175] | Uphill | 2024 | PR | Apple Watch S5 , Garmin Forerunner 935 | vigorous | 50 | 0 | 30.9 ± 4.6 | NA | 87.3 ± 7.7 | 181 ± 6.4 | NA |
| [176] | Van Oost | 2024 | PR | Garmin Vivosmart 4 , Fitbit Charge 5 , Fitbit Sense 2 , Withings ScanWatch , WHOOP 4 | rest , light , moderate , vigorous | 24 | 58.3 | 23.2 ± 1.7 | NA | NA | NA | NA |
| [177] | Vermunicht | 2025 | PR | Fitbit Inspire 2 | mixed | 15 | 13.3 | NA | NA | NA | NA | NA |
| [178] | Wallen | 2016 | PR | Apple Watch , Fitbit Charge HR , Samsung Gear S , Mio Alpha | rest , light , moderate , vigorous | 22 | 50 | 24.9 ± 5.6 | NA | 72.7 ± 11.8 | 173.1 ± 9.9 | NA |
| [179] | Walzel | 2023 | SpO_2_ | Apple Watch S8 , Samsung Galaxy Watch 5 , Withings ScanWatch | rest | 18 | 22.2 | 23.2 ± 1.8 | 24.6 ± 3.2 | NA | NA | NA |
| [180] | Wang | 2017 | PR | Fitbit Charge HR , Apple Watch , Mio Alpha , Basis Peak | rest , moderate , vigorous | 50 | 56 | 37 ± 11.3 | 23.5 ± 3.1 | NA | NA | NA |
| [181] | Weis | 2024 | SpO_2_ | Apple Watch S7 | rest , light | 36 | 44.4 | NA | NA | NA | NA | NA |
| [182] | Williams | 2023 | PR, HRV, SpO_2_ | Garmin Venu 2S | rest | 27 | 37 | 21.9 ± 6.7 | NA | NA | NA | NA |
| [183] | Wolf | 2024 | PR | Apple Watch Ultra , Garmin Enduro 2 | moderate , vigorous | 10 | 40 | 32.8 ± 15.03 | NA | 68.2 ± 15.73 | 170.8 ± 9.13 | NA |
| [184] | Yang | 2025 | PR | Polar Verity sense , Polar Vantage V | moderate , vigorous | 28 | 42.9 | 20.39 ± 1.18 | 21.28 ± 1.71 | 65.06 ± 9.19 | 174 ± 8 | NA |
| [185] | Yasuda | 2025 | PR | Apple Watch | rest | 213 | 37.6 | NA | NA | NA | NA | NA |
| [186] | Yi | 2022 | BP | Huawei Watch D | rest , light , moderate | 85 | 43.5 | 37 ± 14 | NA | NA | NA | NA |
| [187] | Yu | 2023 | PR | Polar OH1 | mixed | 16 | 56.2 | 42 | NA | NA | NA | NA |

1.	Abdalazim N, Larraza JAA, Alchieri L, Alecci L, Santini S, Gashi S, editors. Heart Rate During Sleep Measured Using Finger-, Wrist- and Chest-Worn Devices: A Comparison Study. Pervasive Computing Technologies for Healthcare; 2023 2023//; Cham: Springer Nature Switzerland.

2.	AbiMansour JP, Kaur J, Velaga S, Vatsavayi P, Vogt M, Chandrasekhara V. Accuracy and role of consumer facing wearable technology for continuous monitoring during endoscopic procedures. Frontiers in Digital Health. 2024 2024;6. PMID: rayyan-289033814. doi: doi:10.3389/fdgth.2024.1422929.

3.	Abt G, Bray J, Benson AC. The validity and inter-device variability of the Apple Watch™ for measuring maximal heart rate. Journal of Sports Sciences. 2018 2018;36(13):1447-52. PMID: rayyan-14776683. doi: doi:10.1080/02640414.2017.1397282.

4.	Ahn J, Ra HK, Yoon HJ, Son SH, Ko J. On-Device Filter Design for Self-Identifying Inaccurate Heart Rate Readings on Wrist-Worn PPG Sensors. IEEE Access. 2020 2020;8:184774-84. PMID: rayyan-14776689. doi: doi:10.1109/ACCESS.2020.3025776.

5.	Alfonso C, Garcia-Gonzalez MA, Parrado E, Gil-Rojas J, Ramos-Castro J, Capdevila L. Agreement between two photoplethysmography-based wearable devices for monitoring heart rate during different physical activity situations: a new analysis methodology. Scientific Reports. 2022 2022;12(1). PMID: rayyan-14776699. doi: doi:10.1038/s41598-022-18356-9.

6.	Al-Kaisey AM, Koshy AN, Ha FJ, Spencer R, Toner L, Sajeev JK, et al. Accuracy of wrist-worn heart rate monitors for rate control assessment in atrial fibrillation. International Journal of Cardiology. 2020 2020;300:161-4. PMID: rayyan-14776696. doi: doi:10.1016/j.ijcard.2019.11.120.

7.	Antognoli L, Panni L, Cosoli G, Scalise L. Metrological Characterization of Commercial Smartwatches: are these Sensors Suitable for the Assessment of Well-being? Università Politecnica Delle Marche, Dept. of Industrial Engineering and Mathematical Sciences, Ancona, Italy: Institute of Electrical and Electronics Engineers Inc.; 2023 2023. 6-10 p. ISBN: 9781665456937 (ISBN).

8.	Arslan B, Sener K, Guven R, Kapci M, Korkut S, Sutasir MN, et al. Accuracy of the Apple Watch in measuring oxygen saturation: comparison with pulse oximetry and ABG. Irish Journal of Medical Science. 2024 2024;193(1):477-83. PMID: rayyan-14776714. doi: doi:10.1007/s11845-023-03456-w.

9.	Baek S, Ha Y, Park HW. Accuracy of Wearable Devices for Measuring Heart Rate During Conventional and Nordic Walking. PM and R. 2021 2021;13(4):379-86. PMID: rayyan-14776717. doi: doi:10.1002/pmrj.12424.

10.	Bai Y, Hibbing P, Mantis C, Welk GJ. Comparative evaluation of heart rate-based monitors: Apple Watch vs Fitbit Charge HR. Journal of Sports Sciences. 2018 2018;36(15):1734-41. PMID: rayyan-14776719. doi: doi:10.1080/02640414.2017.1412235.

11.	Bai Y, Tompkins C, Gell N, Dione D, Zhang T, Byun W. Comprehensive comparison of Apple Watch and Fitbit monitors in a free-living setting. PLoS ONE. 2021 2021;16(5). PMID: rayyan-14776720. doi: doi:10.1371/journal.pone.0251975.

12.	Barrios L, Oldrati P, Santini S, Lutterotti A, The European Alliance for I. Evaluating the accuracy of heart rate sensors based on photoplethysmography for in-the-wild analysis. Department of Computer Science, ETH Zurich, Zurich, Switzerland Faculty of Informatics, Università della Svizzera Italiana (USI), Lugano, Switzerland Neuroimmunology and Multiple Sclerosis Research Department of Neurology, University of Zurich, Zurich, Switzerland: ICST; 2019 2019. 251-61 p. ISBN: 21531633 (ISSN); 9781450361262 (ISBN).

13.	Benedetti D, Olcese U, Frumento P, Bazzani A, Bruno S, d'Ascanio P, et al. Heart rate detection by Fitbit ChargeHR(™) : A validation study versus portable polysomnography. J Sleep Res. 2021 2021-12;30(6):e13346. PMID: rayyan-14776730. doi: doi:10.1111/jsr.13346.

14.	Benedetto S, Caldato C, Bazzan E, Greenwood DC, Pensabene V, Actis P. Assessment of the fitbit charge 2 for monitoring heart rate. PLoS ONE. 2018 2018;13(2). PMID: rayyan-14776732. doi: doi:10.1371/journal.pone.0192691.

15.	Bent B, Goldstein BA, Kibbe WA, Dunn JP. Investigating sources of inaccuracy in wearable optical heart rate sensors. npj Digital Medicine. 2020 2020;3(1). PMID: rayyan-14776733. doi: doi:10.1038/s41746-020-0226-6.

16.	Bonneval L, Wing D, Sharp S, Tristao Parra M, Moran R, LaCroix A, et al. Validity of Heart Rate Variability Measured with Apple Watch Series 6 Compared to Laboratory Measures. Sensors. 2025 2025;25(8). PMID: rayyan-289033852. doi: doi:10.3390/s25082380.

17.	Boudreaux BD, Hebert EP, Hollander DB, Williams BM, Cormier CL, Naquin MR, et al. Validity of Wearable Activity Monitors during Cycling and Resistance Exercise. Medicine and Science in Sports and Exercise. 2018 2018;50(3):624-33. PMID: rayyan-14776739. doi: doi:10.1249/MSS.0000000000001471.

18.	Brazendale K, Decker L, Hunt ET, Perry MW, Brazendale AB, Weaver RG, et al. Validity and Wearability of Consumer-based Fitness Trackers in Free-living Children. International Journal of Exercise Science. 2019 2019;12(5):471-82. PMID: rayyan-14776740. doi: doi:.

19.	Bretonneau Q, Peruque-Gayou E, Wolfs E, Bosquet L. Parameters Influencing the Accuracy of a Wrist Photoplethysmography Heart-Rate Monitor (Polar Unite) During Exercise. International Journal of Sports Physiology and Performance. 2023 2023;18(4):440-3. PMID: rayyan-14776741. doi: doi:10.1123/ijspp.2022-0288.

20.	Bretonneau Q, Peruque-Gayou E, Wolfs E, Bosquet L. Accuracy of Heart-Rate-Recovery Parameters Assessed From a Wrist-Worn Photoplethysmography Monitor (Polar Unite). International Journal of Sports Physiology and Performance. 2024 2024;19(1):13-8. PMID: rayyan-14776742. doi: doi:10.1123/ijspp.2023-0120.

21.	Browne SH, Vaida F, Umlauf A, Kim J, DeYoung P, Owens RL. Performance of a commercial smart watch compared to polysomnography reference for overnight continuous oximetry measurement and sleep apnea evaluation. J Clin Sleep Med. 2024 2024-4-23. PMID: rayyan-14776743. doi: doi:10.5664/jcsm.11178.

22.	Budig M, Keiner M, Stoohs R, Hoffmeister M, Höltke V. Heart Rate and Distance Measurement of Two Multisport Activity Trackers and a Cellphone App in Different Sports: A Cross-Sectional Validation and Comparison Field Study. Sensors (Basel). 2021 2021-12-28;22(1). PMID: rayyan-14776746. doi: doi:10.3390/s22010180.

23.	Budig M, Stoohs R, Keiner M. Validity of Two Consumer Multisport Activity Tracker and One Accelerometer against Polysomnography for Measuring Sleep Parameters and Vital Data in a Laboratory Setting in Sleep Patients. Sensors. 2022 2022;22(23). PMID: rayyan-14776748. doi: doi:10.3390/s22239540.

24.	Cadmus-Bertram L, Gangnon R, Wirkus EJ, Thraen-Borowski KM, Gorzelitz-Liebhauser J. The Accuracy of Heart Rate Monitoring by Some Wrist-Worn Activity Trackers. Annals of Internal Medicine. 2017 2017/04/18;166(8):610-2. doi: 10.7326/L16-0353.

25.	Cao R, Azimi I, Sarhaddi F, Niela-Vilen H, Axelin A, Liljeberg P, et al. Accuracy Assessment of Oura Ring Nocturnal Heart Rate and Heart Rate Variability in Comparison With Electrocardiography in Time and Frequency Domains: Comprehensive Analysis. Journal of Medical Internet Research. 2022 2022;24(1). PMID: rayyan-14776753. doi: doi:10.2196/27487.

26.	Carrier B, Marten Chaves S, Navalta JW. Validation of Aerobic Capacity (VO2max) and Pulse Oximetry in Wearable Technology. Sensors. 2025 2025;25(1). PMID: rayyan-289033865. doi: doi:10.3390/s25010275.

27.	Ceugniez M, Devanne H, Hermand E. Reliability and Accuracy of the Fitbit Charge 4 Photoplethysmography Heart Rate Sensor in Ecological Conditions: Validation Study. JMIR mHealth and uHealth. 2025 2025;13. PMID: rayyan-289033868. doi: doi:10.2196/54871.

28.	Chase SE, Liddell RG, McGonagle CL, Ives SJ. Accuracy of the Apple Watch Series 9 for Measures of Energy Expenditure and Heart Rate at Rest and During Exercise: Impact of Skin Pigmentation. Journal of Functional Morphology and Kinesiology. 2024 2024;9(4). PMID: rayyan-289033872. doi: doi:10.3390/jfmk9040275.

29.	Choi JH, Kim HJ. Accuracy Verification of Heart Rate and Energy Consumption Tracking Devices to Develop Forest-Based Customized Health Care Service Programs. Journal of People, Plants, and Environment. 2019 2019;22(2):219-29. PMID: rayyan-14776774. doi: doi:10.11628/ksppe.2019.22.2.219.

30.	Choksatchawathi T, Ponglertnapakorn P, Ditthapron A, Leelaarporn P, Wisutthisen T, Piriyajitakonkij M, et al. Improving Heart Rate Estimation on Consumer Grade Wrist-Worn Device Using Post-Calibration Approach. IEEE Sensors Journal. 2020;20(13):7433-46. doi: 10.1109/JSEN.2020.2979191.

31.	Chow HW, Yang CC. Accuracy of optical heart rate sensing technology in wearable fitness trackers for young and older adults: Validation and comparison study. JMIR mHealth and uHealth. 2020 2020;8(4). PMID: rayyan-14776779. doi: doi:10.2196/14707.

32.	Claes J, Buys R, Avila A, Finlay D, Kennedy A, Guldenring D, et al. Validity of heart rate measurements by the Garmin Forerunner 225 at different walking intensities. Journal of Medical Engineering and Technology. 2017 2017;41(6):480-5. PMID: rayyan-14776781. doi: doi:10.1080/03091902.2017.1333166.

33.	Climstein M, Alder JL, Brooker AM, Cartwright EJ, Kemp-Smith K, Simas V, et al. Reliability of the Polar Vantage M Sports Watch when Measuring Heart Rate at Different Treadmill Exercise Intensities. Sports (Basel). 2020 2020-8-23;8(9). PMID: rayyan-14776786. doi: doi:10.3390/sports8090117.

34.	Coelli S, Carrara M, Bianchi AM, De Tommaso M, Actis-Grosso R, Reali P, editors. Comparing Consumer and Research-Grade Wristbands for Inter-Beat Intervals Monitoring. 2024 2024: Institute of Electrical and Electronics Engineers Inc.

35.	Collins T, Woolley SI, Oniani S, Pires IM, Garcia NM, Ledger SJ, et al. Version Reporting and Assessment Approaches for New and Updated Activity and Heart Rate Monitors. Sensors [Internet]. 2019; 19(7).

36.	Cosoli G, Antognoli L, Scalise L, Instrumentation I, Measurement S, Chapter ISCI, et al. Indirect Estimation of Breathing Rate through Wearable Devices. Marche Polytechnic University, Dept. of Industrial Eng. & Mathematical Sciences, Ancona, Italy: Institute of Electrical and Electronics Engineers Inc.; 2022 2022. ISBN: 9781665482998 (ISBN).

37.	Cosoli G, Antognoli L, Scalise L. Wearable Electrocardiography for Physical Activity Monitoring: Definition of Validation Protocol and Automatic Classification. Biosensors. 2023 2023;13(2). PMID: rayyan-14776793. doi: doi:10.3390/bios13020154.

38.	Cosoli G, Antognoli L, Veroli V, Scalise L. Accuracy and Precision ofWearable Devices for Real-Time Monitoring of Swimming Athletes. Sensors. 2022 2022;22(13). PMID: rayyan-14776794. doi: doi:10.3390/s22134726.

39.	Danielsson ML, Vergeer M, Plasqui G, Baumgart JK. Accuracy of the Apple Watch Series 4 and Fitbit Versa for Assessing Energy Expenditure and Heart Rate of Wheelchair Users During Treadmill Wheelchair Propulsion: Cross-sectional Study. JMIR Formative Research. 2024 2024;8. PMID: rayyan-14776801. doi: doi:10.2196/52312.

40.	De Oliveira Damasceno V, Dos Santos Costa A, Campello MCA, Souza DE, Gonçalves R, Campos EZ, et al. Criterion validity and accuracy of a heart rate monitor. Human Movement. 2022 2022;23(1):60-8. PMID: rayyan-14776804. doi: doi:10.5114/hm.2021.104188.

41.	de Zambotti M, Baker FC, Willoughby AR, Godino JG, Wing D, Patrick K, et al. Measures of sleep and cardiac functioning during sleep using a multi-sensory commercially-available wristband in adolescents. Physiology and Behavior. 2016 2016;158:143-9. PMID: rayyan-14776805. doi: doi:10.1016/j.physbeh.2016.03.006.

42.	Dondzila CJ, Lewis C, Lopez JR, Parker T. Congruent accuracy of wrist-worn activity trackers during controlled and free-living conditions. International journal of exercise science. 2018;11(7):575-84.

43.	Dooley EE, Golaszewski NM, Bartholomew JB. Estimating accuracy at exercise intensities: A comparative study of self-monitoring heart rate and physical activity wearable devices. JMIR mHealth and uHealth. 2017 2017;5(3). PMID: rayyan-14776813. doi: doi:10.2196/mhealth.7043.

44.	Düking P, Giessing L, Frenkel MO, Koehler K, Holmberg HC, Sperlich B. Wrist-worn wearables for monitoring heart rate and energy expenditure while sitting or performing light-to-vigorous physical activity: Validation study. JMIR mHealth and uHealth. 2020 2020;8(5). PMID: rayyan-14776816. doi: doi:10.2196/16716.

45.	El-Amrawy F, Nounou MI. Are Currently Available Wearable Devices for Activity Tracking and Heart Rate Monitoring Accurate, Precise, and Medically Beneficial? Healthc Inform Res. 2015 10;21(4):315-20. doi: 10.4258/hir.2015.21.4.315.

46.	Etiwy M, Akhrass Z, Gillinov L, Alashi A, Wang R, Blackburn G, et al. Accuracy of wearable heart rate monitors in cardiac rehabilitation. Cardiovasc Diagn Ther. 2019 2019-6;9(3):262-71. PMID: rayyan-14776827. doi: doi:10.21037/cdt.2019.04.08.

47.	Falter M, Budts W, Goetschalckx K, Cornelissen V, Buys R. Accuracy of apple watch measurements for heart rate and energy expenditure in patients with cardiovascular disease: Cross-sectional study. JMIR mHealth and uHealth. 2019 2019;7(3). PMID: rayyan-14776831. doi: doi:10.2196/11889.

48.	Falter M, Scherrenberg M, Driesen K, Pieters Z, Kaihara T, Xu L, et al. Smartwatch-Based Blood Pressure Measurement Demonstrates Insufficient Accuracy. Frontiers in Cardiovascular Medicine. 2022 2022;9. PMID: rayyan-14776832. doi: doi:10.3389/fcvm.2022.958212.

49.	Ferrera LE, Caballero JM, Kurniawan Y, Tedjasaputra JA, Sari E, moz:lla. Health quadrant mhealth application: A performance analysis of the heart rate accuracy vis a vis a pulse oximeter. Technological Institute of the Philippines, Manila, Philippines: Association for Computing Machinery; 2018 2018. 54-60 p. ISBN: 9781450364294 (ISBN).

50.	Flett IL, Su Y, Cameron C, Zhou C, Pearson LH, Chase JG, et al., editors. Assessing Accuracy of a Wrist-Worn Wearable Device over Changing Levels of Activity. 2024 2024: Elsevier B.V.

51.	Gagnon J, Khau M, Lavoie-Hudon L, Vachon F, Drapeau V, Tremblay S. Comparing a Fitbit Wearable to an Electrocardiogram Gold Standard as a Measure of Heart Rate under Psychological Stress: A Validation Study. JMIR Formative Research. 2022 2022;6(12). PMID: rayyan-14776849. doi: doi:10.2196/37885.

52.	Gaynor M, Sawyer A, Jenkins S, Wood J. Variable agreement between wearable heart rate monitors during exercise in cystic fibrosis. ERJ Open Research. 2019 2019;5(4). PMID: rayyan-14776852. doi: doi:10.1183/23120541.00006-2019.

53.	Ge Z, Prasad PWC, Costadopoulos N, Alsadoon A, Singh AK, Elchouemi A, et al. Evaluating the accuracy of wearable heart rate monitors. Charles Sturt University Study Centre, Sydney, Australia Department of Computer Applications, National Institute of Technology, Haryana, India Walden University, United States: Institute of Electrical and Electronics Engineers Inc.; 2016 2016. ISBN: 9781509034802 (ISBN).

54.	Giggins OM, Doyle J, Sojan N, Moran O, Crabtree DR, Fraser M, et al. Accuracy of Wrist-Worn Photoplethysmography Devices at Measuring Heart Rate in the Laboratory and during Free-Living Activities. Netwell CASALA, Dundalk Institute of Technology, Dundalk, Co. Louth, Ireland Centre for Health Science, Division of Biomedical Sciences, University of the Highlands and Islands, Old Perth Road, Inverness, IV2 3JH, United Kingdom School of Applied Sciences, Edinburgh Napier University, Edinburgh, United Kingdom: Institute of Electrical and Electronics Engineers Inc.; 2021 2021. 6970-3 p. ISBN: 1557170X (ISSN); 9781728111797 (ISBN).

55.	Gilgen-Ammann R, Roos L, Wyss T, Veenstra BJ, Delves SK, Beeler N, et al. Validation of ambulatory monitoring devices to measure energy expenditure and heart rate in a military setting. Physiological Measurement. 2021 2021;42(8). PMID: rayyan-14776860. doi: doi:10.1088/1361-6579/ac19f9.

56.	Gillinov S, Etiwy M, Wang R, Blackburn G, Phelan D, Gillinov AM, et al. Variable accuracy of wearable heart rate monitors during aerobic exercise. Medicine and Science in Sports and Exercise. 2017 2017;49(8):1697-703. PMID: rayyan-14776862. doi: doi:10.1249/MSS.0000000000001284.

57.	Gorny AW, Liew SJ, Tan CS, Müller-Riemenschneider F. Fitbit charge HR wireless heart rate monitor: Validation study conducted under free-living conditions. JMIR mHealth and uHealth. 2017 2017;5(10). PMID: rayyan-14776870. doi: doi:10.2196/mhealth.8233.

58.	Haghayegh S, Khoshnevis S, Smolensky MH, Diller KR. Accuracy of PurePulse photoplethysmography technology of Fitbit Charge 2 for assessment of heart rate during sleep. Chronobiology International. 2019 2019. PMID: rayyan-14776878. doi: doi:10.1080/07420528.2019.1596947.

59.	Hahnen C, Freeman CG, Haldar N, Hamati JN, Bard DM, Murali V, et al. Accuracy of Vital Signs Measurements by a Smartwatch and a Portable Health Device: Validation Study. JMIR Mhealth Uhealth. 2020 2020/2/12;8(2):e16811. doi: 10.2196/16811.

60.	Hänsel K, Poguntke R, Haddadi H, Alomainy A, Schmidt A, Acm S. What to put on the user: Sensing technologies for studies and physiology aware systems. Queen Mary University of London, London, United Kingdom University of Stuttgart, London, Germany Imperial College, London, United Kingdom Ludwig Maximilian University, Munich, Germany: Association for Computing Machinery; 2018 2018. ISBN: 9781450356206 (ISBN); 9781450356213 (ISBN).

61.	Haveman ME, van Rossum MC, Vaseur RME, van der Riet C, Schuurmann RCL, Hermens HJ, et al. Continuous Monitoring of Vital Signs With Wearable Sensors During Daily Life Activities: Validation Study. JMIR Form Res. 2022 2022-1-7;6(1):e30863. PMID: rayyan-14776889. doi: doi:10.2196/30863.

62.	Held NJ, Perrotta AS, Mueller T, Pfoh-MacDonald SJ. Agreement of the Apple Watch® and Fitbit Charge® for recording step count and heart rate when exercising in water. Medical & Biological Engineering & Computing. 2022 2022/05/01;60(5):1323-31. doi: 10.1007/s11517-022-02536-w.

63.	Helmer P, Hottenrott S, Rodemers P, Leppich R, Helwich M, Pryss R, et al. Accuracy and Systematic Biases of Heart Rate Measurements by Consumer-Grade Fitness Trackers in Postoperative Patients: Prospective Clinical Trial. Journal of Medical Internet Research. 2022 2022;24(12). PMID: rayyan-14776894. doi: doi:10.2196/42359.

64.	Helmer P, Hottenrott S, Wienböker K, Pryss R, Drosos V, Seitz AK, et al. Reliability of continuous vital sign monitoring in post-operative patients employing consumer-grade fitness trackers: A randomised pilot trial. Digital Health. 2024 2024;10. PMID: rayyan-14776895. doi: doi:10.1177/20552076241254026.

65.	Hermand E, Coll C, Richalet JP, Lhuissier FJ. Accuracy and Reliability of Pulse O 2Saturation Measured by a Wrist-worn Oximeter. International Journal of Sports Medicine. 2021 2021;42(14):1268-73. PMID: rayyan-14776899. doi: doi:10.1055/a-1337-2790.

66.	Hermans F, Arents E, Blondeel A, Janssens W, Cardinaels N, Calders P, et al. Validity of a Consumer-Based Wearable to Measure Clinical Parameters in Patients With Chronic Obstructive Pulmonary Disease and Healthy Controls: Observational Study. JMIR mHealth and uHealth. 2024 2024;12. PMID: rayyan-289033970. doi: doi:10.2196/56027.

67.	Hernando D, Roca S, Sancho J, Alesanco Á, Bailón R. Validation of the apple watch for heart rate variability measurements during relax and mental stress in healthy subjects. Sensors (Switzerland). 2018 2018;18(8). PMID: rayyan-14776901. doi: doi:10.3390/s18082619.

68.	Hettiarachchi IT, Hanoun S, Nahavandi D, Nahavandi S. Validation of Polar OH1 optical heart rate sensor for moderate and high intensity physical activities. PLOS ONE. 2019;14(5):e0217288. doi: 10.1371/journal.pone.0217288.

69.	Ho WT, Yang YJ, Li TC. Accuracy of wrist-worn wearable devices for determining exercise intensity. Digital Health. 2022 2022;8. PMID: rayyan-14776908. doi: doi:10.1177/20552076221124393.

70.	Hoevenaars D, Yocarini IE, Paraschiakos S, Holla JFM, de Groot S, Kraaij W, et al. Accuracy of Heart Rate Measurement by the Fitbit Charge 2 During Wheelchair Activities in People With Spinal Cord Injury: Instrument Validation Study. JMIR Rehabilitation and Assistive Technologies. 2022 2022;9(1). PMID: rayyan-14776909. doi: doi:10.2196/27637.

71.	Horton JF, Stergiou PRO, Fung TS, Katz L. Comparison of Polar M600 Optical Heart Rate and ECG Heart Rate during Exercise. Medicine & Science in Sports & Exercise. 2017;49(12).

72.	Hough P, Glaister M, Pledger A. The Accuracy of Wrist-worn Heart Rate Monitors across a Range of Exercise Intensities. Journal of Physical Activity Research. 2017 2017/11/25;2(2):112-6. doi: 10.12691/jpar-2-2-8.

73.	Hung SH, Serwa K, Rosenthal G, Eng JJ. Validity of heart rate measurements in wrist-based monitors across skin tones during exercise. PLoS ONE. 2025 2025;20(2). PMID: rayyan-289033990. doi: doi:10.1371/journal.pone.0318724.

74.	Huynh P, Shan R, Osuji N, Ding J, Isakadze N, Marvel FA, et al. Heart rate measurements in patients with obstructive sleep apnea and atrial fibrillation: Prospective pilot study assessing Apple Watch’s agreement with telemetry data. JMIR Cardio. 2021 2021;5(1). PMID: rayyan-14776914. doi: doi:10.2196/18050.

75.	Hwang J, Kim J, Choi KJ, Cho MS, Nam GB, Kim YH. Assessing accuracy of wrist-worn wearable devices in measurement of paroxysmal supraventricular tachycardia heart rate. Korean Circulation Journal. 2019 2019;49(5):437-45. PMID: rayyan-14776916. doi: doi:10.4070/KCJ.2018.0323.

76.	Ibrahim NS, Rampal S, Lee WL, Pek EW, Suhaimi A. Evaluation of Wrist-Worn Photoplethysmography Trackers with an Electrocardiogram in Patients with Ischemic Heart Disease: A Validation Study. Cardiovascular Engineering and Technology. 2024 2024/02/01;15(1):12-21. doi: 10.1007/s13239-023-00693-z.

77.	Icenhower A, Murphy C, Brooks AK, Irby M, N'Dah K, Robison J, et al. Investigating the accuracy of Garmin PPG sensors on differing skin types based on the Fitzpatrick scale: cross-sectional comparison study. Front Digit Health. 2025 2025;7:1553565. PMID: rayyan-289033994. doi: doi:10.3389/fdgth.2025.1553565.

78.	Imai H, Shimada J. Evaluation of Telemetric Single-Lead Cardiac Transmitter and Apple Watch for Heart Rate Monitoring: Implications for Heart Failure Management in Home Care. Cureus. 2025 2025-3;17(3):e80232. PMID: rayyan-289033998. doi: doi:10.7759/cureus.80232.

79.	Jachymek M, Jachymek MT, Kiedrowicz RM, Kaźmierczak J, Płońska-Gościniak E, Peregud-Pogorzelska M. Wristbands in Home-Based Rehabilitation-Validation of Heart Rate Measurement. Sensors (Basel). 2021 2021-12-23;22(1). PMID: rayyan-14776919. doi: doi:10.3390/s22010060.

80.	Jaiswal P, Sahu NK, Lone HR. Comparative Assessment of Smartwatch Photoplethysmography Accuracy. IEEE Sensors Letters. 2024;8(1):1-4. doi: 10.1109/LSENS.2023.3342292.

81.	Jamieson A, Jones S, Chaturvedi N, Hughes AD, Orini M. Accuracy of smartwatches for the remote assessment of exercise capacity. Scientific Reports. 2024 2024;14(1). PMID: rayyan-289034010. doi: doi:10.1038/s41598-024-74140-x.

82.	Jarchi D, Salvi D, Velardo C, Mahdi A, Tarassenko L, Clifton DA, et al. Estimation of HRV and SpO2 from wrist-worn commercial sensors for clinical settings. Engineering Science Department, University of Oxford, Oxford, OX3 7DQ, United Kingdom: Institute of Electrical and Electronics Engineers Inc.; 2018 2018. 144-7 p. ISBN: 9781538611098 (ISBN).

83.	Jiang Y, Spies C, Magin J, Bhosai SJ, Snyder L, Dunn J. Investigating the accuracy of blood oxygen saturation measurements in common consumer smartwatches. PLOS Digit Health. 2023 2023-7;2(7):e0000296. PMID: rayyan-14776930. doi: doi:10.1371/journal.pdig.0000296.

84.	Jo E, Lewis K, Directo D, Kim MJ, Dolezal BA. Validation of Biofeedback Wearables for Photoplethysmographic Heart Rate Tracking. J Sports Sci Med. 2016 2016-9;15(3):540-7. PMID: rayyan-14776931. doi: doi:.

85.	Jørgensen A, Toftager M, Eghøj M, Ried-Larsen M, Bjørk Petersen C. Heart rate responses, agreement and accuracy among persons with severe disabilities participating in the indirect movement program: Team Twin—an observational study. Frontiers in Sports and Active Living. 2023 2023;5. PMID: rayyan-14776933. doi: doi:10.3389/fspor.2023.1213655.

86.	Jung H, Kim D, Choi J, Joo EY. Validating a Consumer Smartwatch for Nocturnal Respiratory Rate Measurements in Sleep Monitoring. Sensors. 2023 2023;23(18). PMID: rayyan-14776936. doi: doi:10.3390/s23187976.

87.	Jung H, Kim D, Lee W, Seo H, Seo J, Choi J, et al. Performance evaluation of a wrist-worn reflectance pulse oximeter during sleep. Sleep Health. 2022 2022;8(5):420-8. PMID: rayyan-14776937. doi: doi:10.1016/j.sleh.2022.04.003.

88.	Jung HC, Kang M, Lee NH, Jeon S, Lee S. Impact of Placement of Fitbit HR under Laboratory and Free-Living Conditions. Sustainability [Internet]. 2020; 12(16).

89.	Kang S, Kim Y, Byun W, Suk J, Lee JM. Comparison of a wearable tracker with actigraph for classifying physical activity intensity and heart rate in children. International Journal of Environmental Research and Public Health. 2019 2019;16(15). PMID: rayyan-14776943. doi: doi:10.3390/ijerph16152663.

90.	Khushhal A, Nichols S, Evans W, Gleadall-Siddall DO, Page R, O'Doherty AF, et al. Validity and Reliability of the Apple Watch for Measuring Heart Rate During Exercise. Sports Med Int Open. 2017 2017-10;1(6):E206-e11. PMID: rayyan-14776952. doi: doi:10.1055/s-0043-120195.

91.	Khushhal AA, Mohamed AA, Elsayed ME. Accuracy of Apple Watch to Measure Cardiovascular Indices in Patients with Chronic Diseases: A Cross Sectional Study. Journal of Multidisciplinary Healthcare. 2024 2024;17:1053-63. PMID: rayyan-14776953. doi: doi:10.2147/JMDH.S449071.

92.	Kim C, Song JH, Kim SH. Validation of Wearable Digital Devices for Heart Rate Measurement During Exercise Test in Patients With Coronary Artery Disease. Annals of Rehabilitation Medicine. 2023 2023;47(4):261-71. PMID: rayyan-14776954. doi: doi:10.5535/arm.23019.

93.	Kinnunen H, Rantanen A, Kentt T, Koskim ki H. Feasible assessment of recovery and cardiovascular health: Accuracy of nocturnal HR and HRV assessed via ring PPG in comparison to medical grade ECG. Physiological Measurement. 2020 2020;41(4). PMID: rayyan-14776958. doi: doi:10.1088/1361-6579/ab840a.

94.	Kondama Reddy R, Pooni R, Zaharieva DP, Senf B, El Youssef J, Dassau E, et al. Accuracy of wrist-worn activity monitors during common daily physical activities and types of structured exercise: Evaluation study. JMIR mHealth and uHealth. 2018 2018;6(12). PMID: rayyan-14776964. doi: doi:10.2196/10338.

95.	Koshy AN, Sajeev JK, Nerlekar N, Brown AJ, Rajakariar K, Zureik M, et al. Smart watches for heart rate assessment in atrial arrhythmias. International Journal of Cardiology. 2018 2018;266:124-7. PMID: rayyan-14776965. doi: doi:10.1016/j.ijcard.2018.02.073.

96.	Kozak D, Burgermeister S, Buretel De Chassey JD, Naef A, Maringue A, Dietrich D. A functionality, safety and validity study of innovative REACH devices. Gerontechnology. 2017 2017;16(3):181-8. PMID: rayyan-14776966. doi: doi:10.4017/gt.2017.16.3.007.00.

97.	Kroll RR, Boyd JG, Maslove DM. Accuracy of a Wrist-Worn Wearable Device for Monitoring Heart Rates in Hospital Inpatients: A Prospective Observational Study. J Med Internet Res. 2016 2016/09/20;18(9):e253. doi: 10.2196/jmir.6025.

98.	Lauterbach CJ, Romano PA, Greisler LA, Brindle RA, Ford KR, Kuennen MR. Accuracy and Reliability of Commercial Wrist-Worn Pulse Oximeter During Normobaric Hypoxia Exposure Under Resting Conditions. Research Quarterly for Exercise and Sport. 2020 2020:1-10. PMID: rayyan-14776979. doi: doi:10.1080/02701367.2020.1759768.

99.	Lee H, Lee M, Park S. Accuracy of Wearable Devices for Estimating Energy Expenditure and Heart Rate During Golf. Exercise Science. 2022 2022;31(3):411-21. PMID: rayyan-14776983. doi: doi:10.15857/ksep.2022.00178.

100.	Leth S, Hansen J, Nielsen OW, Dinesen B. Evaluation of commercial self-monitoring devices for clinical purposes: Results from the future patient trial, phase I. Sensors (Switzerland). 2017 2017;17(1). PMID: rayyan-14776994. doi: doi:10.3390/s17010211.

101.	Lima CS, Bertocco FC, Oliveira JIVd, Souza TMFd, Silva EPd, Zuben FJV. Assessment of Samsung Galaxy Watch4 PPG-Based Heart Rate During Light-to-Vigorous Physical Activities. IEEE Sensors Letters. 2024;8(7):1-4. doi: 10.1109/LSENS.2024.3408089.

102.	Lins L, do Nascimento EGC, da Silva Júnior JA, de Medeiros Fernandes TAA, de Andrade MF, de Mesquita Andrade C. Accuracy of wearable electronic device compared to manual and automatic methods of blood pressure determination. Med Biol Eng Comput. 2023 2023-10;61(10):2627-36. PMID: rayyan-14777005. doi: doi:10.1007/s11517-023-02869-0.

103.	Littell L, Roelle L, Dalal A, Van Hare GF, Orr WB, Miller N, et al. Assessment of Apple Watch Series 6 pulse oximetry and electrocardiograms in a pediatric population. PLOS Digit Health. 2022 2022-8;1(8):e0000051. PMID: rayyan-14777007. doi: doi:10.1371/journal.pdig.0000051.

104.	Martín-Escudero P, Cabanas AM, Dotor-Castilla ML, Galindo-Canales M, Miguel-Tobal F, Fernández-Pérez C, et al. Are Activity Wrist-Worn Devices Accurate for Determining Heart Rate during Intense Exercise? Bioengineering. 2023 2023;10(2). PMID: rayyan-14777031. doi: doi:10.3390/bioengineering10020254.

105.	Marzano-Felisatti JM, De Lucca L, Priego-Quesada JI, Pino-Ortega J. Heart Rate Measurement Accuracy During Intermittent Efforts Under Laboratory Conditions: A Comparative Analysis Between Chest Straps and Armband. Applied Sciences (Switzerland). 2024 2024;14(24). PMID: rayyan-289034093. doi: doi:10.3390/app142411872.

106.	Merrigan JJ, Stovall JH, Stone JD, Stephenson M, Finomore VS, Hagen JA. Validation of Garmin and Polar Devices for Continuous Heart Rate Monitoring During Common Training Movements in Tactical Populations. Measurement in Physical Education and Exercise Science. 2023 2023;27(3):234-47. PMID: rayyan-14777037. doi: doi:10.1080/1091367X.2022.2161820.

107.	Meza C, Juega J, Francisco J, Santos A, Duran L, Rodriguez M, et al. Accuracy of a Smartwatch to Assess Heart Rate Monitoring and Atrial Fibrillation in Stroke Patients. Sensors. 2023 2023;23(10). PMID: rayyan-14777039. doi: doi:10.3390/s23104632.

108.	Miller DJ, Sargent C, Roach GD. A Validation of Six Wearable Devices for Estimating Sleep, Heart Rate and Heart Rate Variability in Healthy Adults. Sensors. 2022 2022;22(16). PMID: rayyan-14777041. doi: doi:10.3390/s22166317.

109.	Mink F, Lutz W, Rafaeli E, Hehlmann M, Schwartz B, Uhl J. Validation and Application of Wrist-Worn Wearable Physiological Measures in Psychotherapy. Cognitive Therapy and Research. 2025 2025. PMID: rayyan-289034106. doi: doi:10.1007/s10608-025-10601-5.

110.	Moayedi Y, Abdulmajeed R, Duero Posada J, Foroutan F, Alba AC, Cafazzo J, et al. Assessing the Use of Wrist-Worn Devices in Patients With Heart Failure: Feasibility Study. JMIR Cardio. 2017 2017-12-19;1(2):e8. PMID: rayyan-14777043. doi: doi:10.2196/cardio.8301.

111.	Montalvo S, Martinez A, Arias S, Lozano A, Gonzalez MP, Dietze-Hermosa MS, et al. Commercial Smart Watches and Heart Rate Monitors: A Concurrent Validity Analysis. Journal of Strength and Conditioning Research. 2023 2023;37(9):1802-8. PMID: rayyan-14777046. doi: doi:10.1519/JSC.0000000000004482.

112.	Monteiro R, Rabello GCM, Moreno CR, Moitinho MS, Pires FA, Samesina N, et al. Enhancing cardiac postoperative care: a smartwatch-integrated remote telemonitoring platform for health screening with ECG analysis. Frontiers in Cardiovascular Medicine. 2024 2024;11. PMID: rayyan-289034112. doi: doi:10.3389/fcvm.2024.1443998.

113.	Montes J, Tandy R, Young J, Lee SP, Navalta J. A comparison of multiple wearable technology devices heart rate and step count measurements during free motion and treadmill based measurements. International Journal of Kinesiology and Sports Science. 2019 2019;7(2):30-9. PMID: rayyan-14777047. doi: doi:10.7575/aiac.ijkss.v.7n.2p.30.

114.	Montoye AHK, Mitrzyk JR, Molesky MJ. Comparative Accuracy of a Wrist-Worn Activity Tracker and a Smart Shirt for Physical Activity Assessment. Measurement in Physical Education and Exercise Science. 2017 2017;21(4):201-11. PMID: rayyan-14777048. doi: doi:10.1080/1091367X.2017.1331166.

115.	Muggeridge DJ, Hickson K, Davies AV, Giggins OM, Megson IL, Gorely T, et al. Measurement of heart rate using the polar OH1 and fitbit charge 3 wearable devices in healthy adults during light, moderate, vigorous, and sprint-based exercise: Validation study. JMIR mHealth and uHealth. 2021 2021;9(3). PMID: rayyan-14777053. doi: doi:10.2196/25313.

116.	Müller AM, Wang NX, Yao J, Tan CS, Low ICC, Lim N, et al. Heart Rate Measures From Wrist-Worn Activity Trackers in a Laboratory and Free-Living Setting: Validation Study. JMIR Mhealth Uhealth. 2019 2019/10/2;7(10):e14120. doi: 10.2196/14120.

117.	Nash D, Shah MJ, Shehab O, Jones AL, Iyer R, Vetter V, et al. “But for the blind spot”: Accuracy and diagnostic performance of smart watch cardiac features in pediatric patients. Heart Rhythm. 2024 2024;21(5):581-9. PMID: rayyan-14777061. doi: doi:10.1016/j.hrthm.2024.01.021.

118.	Navalta JW, Davis DW, Carrier B, Malek EM, Vargas N, Rodriguez JP, et al. Validity and Reliability of Wearable Devices during Self-Paced Walking, Jogging and Overground Skipping. Sport Mont. 2023 2023;21(3):23-9. PMID: rayyan-14777063. doi: doi:10.26773/smj.231004.

119.	Navalta JW, Montes J, Bodell NG, Salatto RW, Manning JW, DeBeliso M. Concurrent heart rate validity of wearable technology devices during trail running. PLoS ONE. 2020 2020;15(8). PMID: rayyan-14777065. doi: doi:10.1371/journal.pone.0238569.

120.	Navalta JW, Carrier B, Blank M, Zarei S, Davis DW, Craig M, et al. Validity and Reliability of Wearable Technology Devices during Simulated Pickleball Game Play. Sports. 2024 2024;12(9). PMID: rayyan-289034122. doi: doi:10.3390/sports12090234.

121.	Neudorfer M, Kumar D, Smeddinck JD, Kulnik ST, Niebauer J, Treff G, et al. Validity of Four Consumer-Grade Optical Heart Rate Sensors for Assessing Volume and Intensity Distribution of Physical Activity. Scandinavian Journal of Medicine and Science in Sports. 2024 2024;34(11). PMID: rayyan-289034129. doi: doi:10.1111/sms.14756.

122.	Nissen M, Slim S, Jäger K, Flaucher M, Huebner H, Danzberger N, et al. Heart Rate Measurement Accuracy of Fitbit Charge 4 and Samsung Galaxy Watch Active2: Device Evaluation Study. JMIR Form Res. 2022 2022/3/1;6(3):e33635. doi: 10.2196/33635.

123.	Nuss KJ, Sanford JL, Archambault LJ, Schlemer EJ, Blake S, Courtney JB, et al. Accuracy of Heart Rate and Energy Expenditure Estimations of Wrist-Worn and Arm-Worn Apple Watches. Journal for the Measurement of Physical Behaviour. 2019 2019;2(3):166-75. PMID: rayyan-14777075. doi: doi:10.1123/jmpb.2018-0061.

124.	Nuuttila OP, Korhonen E, Laukkanen J, Kyröläinen H. Validity of the Wrist-Worn Polar Vantage V2 to Measure Heart Rate and Heart Rate Variability at Rest. Sensors (Basel). 2021 2021-12-26;22(1). PMID: rayyan-14777076. doi: doi:10.3390/s22010137.

125.	O’Driscoll R, Turicchi J, Hopkins M, Gibbons C, Larsen SC, Palmeira AL, et al. The validity of two widely used commercial and research-grade activity monitors, during resting, household and activity behaviours. Health and Technology. 2020 2020;10(3):637-48. PMID: rayyan-14777079. doi: doi:10.1007/s12553-019-00392-7.

126.	O'Grady B, Lambe R, Baldwin M, Acheson T, Doherty C. The Validity of Apple Watch Series 9 and Ultra 2 for Serial Measurements of Heart Rate Variability and Resting Heart Rate. Sensors (Basel). 2024 2024-9-26;24(19). PMID: rayyan-289034135. doi: doi:10.3390/s24196220.

127.	Page LL, Fanning J, Phipps C, Berger A, Reed E, Ehlers D. Heart Rate Monitoring Among Breast Cancer Survivors: Quantitative Study of Device Agreement in a Community-Based Exercise Program. JMIR Cancer. 2024 2024;10. PMID: rayyan-289034142. doi: doi:10.2196/51210.

128.	Pasadyn SR, Soudan M, Gillinov M, Houghtaling P, Phelan D, Gillinov N, et al. Accuracy of commercially available heart rate monitors in athletes: A prospective study. Cardiovascular Diagnosis and Therapy. 2019 2019;9(4):379-85. PMID: rayyan-14777089. doi: doi:10.21037/cdt.2019.06.05.

129.	Paslı S, Topçuoğlu H, Yılmaz M, Yadigaroğlu M, İmamoğlu M, Karaca Y. Diagnostic accuracy of apple watch ECG outputs in identifying dysrhythmias: A comparison with 12-Lead ECG in emergency department. American Journal of Emergency Medicine. 2024 2024;79:25-32. PMID: rayyan-14777090. doi: doi:10.1016/j.ajem.2024.01.046.

130.	Passler S, Müller N, Senner V. In-ear pulse rate measurement: A valid alternative to heart rate derived from electrocardiography? Sensors (Switzerland). 2019 2019;19(17). PMID: rayyan-14777091. doi: doi:10.3390/s19173641.

131.	Pätz C, Michaelis A, Markel F, Löffelbein F, Dähnert I, Gebauer RA, et al. Accuracy of the Apple Watch Oxygen Saturation Measurement in Adults and Children with Congenital Heart Disease. Pediatric Cardiology. 2023 2023;44(2):333-43. PMID: rayyan-14777094. doi: doi:10.1007/s00246-022-02987-w.

132.	Pelizzo G, Guddo A, Puglisi A, De Silvestri A, Comparato C, Valenza M, et al. Accuracy of a wrist-worn heart rate sensing device during elective pediatric surgical procedures. Children. 2018 2018;5(3). PMID: rayyan-14777096. doi: doi:10.3390/children5030038.

133.	Pierick AR, Burke KJ, Prusi M, Largent B, Yu S, Lowery RE, et al. Validity of Wrist-Worn Activity Tracker Heart Rate Detection in Fontan Patients during Exercise. Medicine and Science in Sports and Exercise. 2025 2025;57(2):280-4. PMID: rayyan-289034151. doi: doi:10.1249/MSS.0000000000003567.

134.	Pipek LZ, Nascimento RFV, Acencio MMP, Teixeira LR. Comparison of SpO2 and heart rate values on Apple Watch and conventional commercial oximeters devices in patients with lung disease. Scientific Reports. 2021 2021;11(1). PMID: rayyan-14777106. doi: doi:10.1038/s41598-021-98453-3.

135.	Poli A, Cosoli G, Iadarola G, Spinsante S, Scalise L, Instrumentation I, et al. Feasibility of Blood Pressure Measurement through Wearable Devices: Analysis of Smartwatches Performance. Polytechnic University of Marche, Dept. of Information Eng., Ancona, Italy Polytechnic University of Marche, Dept. of Industrial Eng. and Mathematical Sciences, Ancona, Italy: Institute of Electrical and Electronics Engineers Inc.; 2022 2022. ISBN: 9781665482998 (ISBN).

136.	Pope ZC, Lee JE, Zeng N, Gao Z. Validation of four smartwatches in energy expenditure and heart rate assessment during exergaming. Games for Health Journal. 2019 2019;8(3):205-12. PMID: rayyan-14777110. doi: doi:10.1089/g4h.2018.0087.

137.	Powierza CS, Clark MD, Hughes JM, Carneiro KA, Mihalik JP. Validation of a Self-Monitoring Tool for Use in Exercise Therapy. PM and R. 2017 2017;9(11):1077-84. PMID: rayyan-14777111. doi: doi:10.1016/j.pmrj.2017.03.012.

138.	Rafl J, Bachman TE, Rafl-Huttova V, Walzel S, Rozanek M. Commercial smartwatch with pulse oximeter detects short-time hypoxemia as well as standard medical-grade device: Validation study. Digital Health. 2022 2022;8. PMID: rayyan-14777117. doi: doi:10.1177/20552076221132127.

139.	Rajakariar K, Buntine P, Ghaly A, Zhu ZC, Abeygunawardana V, Visakhamoorthy S, et al. Accuracy of Smartwatch Pulse Oximetry Measurements in Hospitalized Patients With Coronavirus Disease 2019. Mayo Clinic Proceedings: Digital Health. 2024 2024;2(1):152-8. PMID: rayyan-289034160. doi: doi:10.1016/j.mcpdig.2024.02.001.

140.	Ravanelli N, Lefebvre K, Brough A, Paquette S, Lin W. Validation of an Open-Source Smartwatch for Continuous Monitoring of Physical Activity and Heart Rate in Adults. Sensors. 2025 2025;25(9). PMID: rayyan-289034163. doi: doi:10.3390/s25092926.

141.	Ray I, Liaqat D, Gabel M, Lara Ed, editors. Skin tone, Confidence, and Data Quality of Heart Rate Sensing in WearOS Smartwatches. 2021 IEEE International Conference on Pervasive Computing and Communications Workshops and other Affiliated Events (PerCom Workshops); 2021 22-26 March 2021.

142.	Reece JD, Bunn JA, Choi M, Navalta JW. Assessing Heart Rate Using Consumer Technology Association Standards. Technologies. 2021 2021;9(3). PMID: rayyan-14777122. doi: doi:10.3390/technologies9030046.

143.	Rho G, Di Rienzo F, Marinai C, Giannetti F, Arcarisi L, Bufano P, et al. Preliminary Assessment of the Samsung Galaxy Watch 5 Accuracy for the Monitoring of Heart Rate and Heart Rate Variability Parameters. Dipartimento Di Ingegneria Dell’Informazione, University of Pisa, Largo Lucio Lazzarino 1, Pisa, 56122, Italy Research Center ”E. Piaggio”, University of Pisa, Largo Lucio Lazzarino 1, Pisa, 56122, Italy Institute of Clinical Physiology, National Research Council, Via Giuseppe Moruzzi 1, Pisa, 56124, Italy: Springer Science and Business Media Deutschland GmbH; 2024 2024. 22-30 p. ISBN: 16800737 (ISSN); 9783031490613 (ISBN).

144.	Rodrigues E, Lima D, Barbosa P, Gonzaga K, Guerra RO, Pimentel M, et al. HRV Monitoring Using Commercial Wearable Devices as a Health Indicator for Older Persons during the Pandemic. Sensors [Internet]. 2022; 22(5).

145.	Rozanski GM, Aqui A, Sivakumaran S, Mansfield A. Consumer wearable devices for activity monitoring among individuals after a stroke: A prospective comparison. JMIR Cardio. 2018 2018;2(1). PMID: rayyan-14777135. doi: doi:10.2196/cardio.8199.

146.	Ruiz-Malagón EJ, Castro-Infantes S, Ritaco-Real M, Soto-Hermoso VM. Concurrent validity of the Polar Precision Prime® photoplethysmographic system to measure heart rate during a tennis training session. Proceedings of the Institution of Mechanical Engineers, Part P: Journal of Sports Engineering and Technology. 2023 2023. PMID: rayyan-14777136. doi: doi:10.1177/17543371231165102.

147.	Ruiz-Malagón EJ, Ruiz-Alias SA, García-Pinillos F, Delgado-García G, Soto-Hermoso VM. Comparison between photoplethysmographic heart rate monitor from Polar Vantage M and Polar V800 with H10 chest strap while running on a treadmill: Validation of the Polar Precision PrimeTM photoplestimographic system. Proceedings of the Institution of Mechanical Engineers, Part P: Journal of Sports Engineering and Technology. 2021 2021;235(3):212-8. PMID: rayyan-14777137. doi: doi:10.1177/1754337120976659.

148.	Saghir N, Aggarwal A, Soneji N, Valencia V, Rodgers G, Kurian T. A comparison of manual electrocardiographic interval and waveform analysis in lead 1 of 12-lead ECG and Apple Watch ECG: A validation study. Cardiovascular Digital Health Journal. 2020 2020;1(1):30-6. PMID: rayyan-14777141. doi: doi:10.1016/j.cvdhj.2020.07.002.

149.	Sañudo B, De Hoyo M, Muñoz-López A, Perry J, Abt G. Pilot Study Assessing the Influence of Skin Type on the Heart Rate Measurements Obtained by Photoplethysmography with the Apple Watch. Journal of Medical Systems. 2019 2019;43(7). PMID: rayyan-14777147. doi: doi:10.1007/s10916-019-1325-2.

150.	Sarhaddi F, Kazemi K, Azimi I, Cao R, Niela-Vilén H, Axelin A, et al. A comprehensive accuracy assessment of Samsung smartwatch heart rate and heart rate variability. PLOS ONE. 2022;17(12):e0268361. doi: 10.1371/journal.pone.0268361.

151.	Schiefer LM, Treff G, Treff F, Schmidt P, Schäfer L, Niebauer J, et al. Validity of peripheral oxygen saturation measurements with the garmin fēnix® 5x plus wearable device at 4559 m. Sensors. 2021 2021;21(19). PMID: rayyan-14777153. doi: doi:10.3390/s21196363.

152.	Schweizer T, Gilgen-Ammann R. Wrist-Worn and Arm-Worn Wearables for Monitoring Heart Rate During Sedentary and Light-to-Vigorous Physical Activities: Device Validation Study. JMIR Cardio. 2025 2025;9. PMID: rayyan-289034188. doi: doi:10.2196/67110.

153.	Sequeira N, D'Souza D, Angaran P, Aves T, Dorian P. Common wearable devices demonstrate variable accuracy in measuring heart rate during supraventricular tachycardia. Heart Rhythm. 2020 2020;17(5):854-9. PMID: rayyan-14777158. doi: doi:10.1016/j.hrthm.2020.02.018.

154.	Seshadri DR, Bittel B, Browsky D, Houghtaling P, Drummond CK, Desai M, et al. Accuracy of the apple watch 4 to measure heart rate in patients with atrial fibrillation. IEEE Journal of Translational Engineering in Health and Medicine. 2020 2020;8. PMID: rayyan-14777159. doi: doi:10.1109/JTEHM.2019.2950397.

155.	Shcherbina A, Mattsson CM, Waggott D, Salisbury H, Christle JW, Hastie T, et al. Accuracy in Wrist-Worn, Sensor-Based Measurements of Heart Rate and Energy Expenditure in a Diverse Cohort. J Pers Med. 2017 2017-5-24;7(2). PMID: rayyan-14777163. doi: doi:10.3390/jpm7020003.

156.	Shumate T, Link M, Furness J, Kemp-Smith K, Simas V, Climstein M. Validity of the Polar Vantage M watch when measuring heart rate at different exercise intensities. PeerJ. 2021 2021;9:e10893. PMID: rayyan-14777169. doi: doi:10.7717/peerj.10893.

157.	Sjoberg V, Westergren J, Monnier A, Lo Martire R, Hagstromer M, Ang BO, et al. Wrist-worn activity trackers in laboratory and free-living settings for patients with chronic pain: Criterion validity study. JMIR mHealth and uHealth. 2021 2021;9(1). PMID: rayyan-14777178. doi: doi:10.2196/24806.

158.	Spaccarotella C, Polimeni A, Mancuso C, Pelaia G, Esposito G, Indolfi C. Assessment of Non-Invasive Measurements of Oxygen Saturation and Heart Rate with an Apple Smartwatch: Comparison with a Standard Pulse Oximeter. Journal of Clinical Medicine. 2022 2022;11(6). PMID: rayyan-14777190. doi: doi:10.3390/jcm11061467.

159.	Spierer DK, Rosen Z, Litman LL, Fujii K. Validation of photoplethysmography as a method to detect heart rate during rest and exercise. Journal of Medical Engineering and Technology. 2015 2015;39(5):264-71. PMID: rayyan-14777192. doi: doi:10.3109/03091902.2015.1047536.

160.	Spinsante S, Porfiri S, Scalise L. Accuracy of Heart Rate Measurements by a Smartwatch in Low Intensity Activities2019 2019-6-28. 1-6 p.

161.	Stahl SE, An HS, Dinkel DM, Noble JM, Lee JM. How accurate are the wrist-based heart rate monitors during walking and running activities? Are they accurate enough? BMJ Open Sport and Exercise Medicine. 2016 2016;2(1). PMID: rayyan-14777194. doi: doi:10.1136/bmjsem-2015-000106.

162.	Stone JD, Ulman HK, Tran K, Thompson AG, Halter MD, Ramadan JH, et al. Assessing the Accuracy of Popular Commercial Technologies That Measure Resting Heart Rate and Heart Rate Variability. Frontiers in Sports and Active Living. 2021 2021;3. PMID: rayyan-14777197. doi: doi:10.3389/fspor.2021.585870.

163.	Støve MP, Graversen AH, Sørensen J. Assessment of Noninvasive Oxygen Saturation in Patients With COPD During Pulmonary Rehabilitation: Smartwatch versus Pulse Oximeter. Respiratory Care. 2023 2023;68(8):1041-8. PMID: rayyan-14777198. doi: doi:10.4187/respcare.10760.

164.	Støve MP, Hansen ECK. Accuracy of the Apple Watch Series 6 and the Whoop Band 3.0 for assessing heart rate during resistance exercises. Journal of Sports Sciences. 2022 2022;40(23):2639-44. PMID: rayyan-14777199. doi: doi:10.1080/02640414.2023.2180160.

165.	Støve MP, Haucke E, Nymann ML, Sigurdsson T, Larsen BT. Accuracy of the wearable activity tracker Garmin Forerunner 235 for the assessment of heart rate during rest and activity. Journal of Sports Sciences. 2019 2019;37(8):895-901. PMID: rayyan-14777200. doi: doi:10.1080/02640414.2018.1535563.

166.	Støve MP, Holm RS, Kjaersgaard AS, Duncker K, Jensen MR, Larsen BT. Measurement latency significantly contributes to reduced heart rate measurement accuracy in wearable devices. Journal of Medical Engineering and Technology. 2020 2020;44(3):125-32. PMID: rayyan-14777201. doi: doi:10.1080/03091902.2020.1753836.

167.	Stucky B, Clark I, Azza Y, Karlen W, Achermann P, Kleim B, et al. Validation of fitbit charge 2 sleep and heart rate estimates against polysomnographic measures in shift workers: naturalistic study. Journal of Medical Internet Research. 2021 2021;23(10). PMID: rayyan-14777204. doi: doi:10.2196/26476.

168.	Tedesco S, Sica M, Ancillao A, Timmons S, Barton J, O'Flynn B. Accuracy of consumer-level and research-grade activity trackers in ambulatory settings in older adults. PLoS One. 2019 2019;14(5):e0216891. PMID: rayyan-14777212. doi: doi:10.1371/journal.pone.0216891.

169.	Theurl F, Schreinlechner M, Sappler N, Toifl M, Dolejsi T, Hofer F, et al. Smartwatch-derived heart rate variability: a head-to-head comparison with the gold standard in cardiovascular disease. European Heart Journal - Digital Health. 2023 2023;4(3):155-64. PMID: rayyan-14777216. doi: doi:10.1093/ehjdh/ztad022.

170.	Thiebaud RS, Funk MD, Patton JC, Massey BL, Shay TE, Schmidt MG, et al. Validity of wrist-worn consumer products to measure heart rate and energy expenditure. Digit Health. 2018 2018-1;4:2055207618770322. PMID: rayyan-14777217. doi: doi:10.1177/2055207618770322.

171.	Thomson EA, Nuss K, Comstock A, Reinwald S, Blake S, Pimentel RE, et al. Heart rate measures from the Apple Watch, Fitbit Charge HR 2, and electrocardiogram across different exercise intensities. Journal of Sports Sciences. 2019 2019;37(12):1411-9. PMID: rayyan-14777219. doi: doi:10.1080/02640414.2018.1560644.

172.	Turki A, Behbehani K, Ding K, Zhang R, Li M, Bell K, et al. Estimation of Heart Rate Variability Measures Using Apple Watch and Evaluating Their Accuracy: Estimation of Heart Rate Variability Measures Using Apple Watch. The University of Texas, Arlington, United States: Association for Computing Machinery; 2021 2021. 565-74 p. ISBN: 9781450387927 (ISBN).

173.	Turki AF, Section II, Universitas Islam I, Universitas Islam I, Chapter ICSI. An Investigation into the Accuracy of Blood Oxygen Saturation Measurement using Smart Watch Devices Among Pediatric Users in Saudi Arabia. King Abdul Aziz University, Center of Excellence in Intelligent Engineering Systems, Electrical and Computer Engineering Department, Jeddah, Saudi Arabia: Institute of Electrical and Electronics Engineers Inc.; 2023 2023. 29-33 p. ISBN: 9798350302424 (ISBN).

174.	Turki AF, Jani MB, Ding K, Zhang R, Behbehani K. An Investigation of Heartrate Sensing Accuracy by Wrist-Worn Fitness Tracking Devices2019 2019-7-27. 3337-40 p. ISBN: 1558-4615.

175.	Uphill A, Kendall KL, Fogarty A, Guppy S, Brown H, Zomer T, et al. Validity of Apple Watch, Garmin Forerunner® 935 and GENEActiv for estimating energy expenditure during close quarter battle training in Special Forces soldiers. European Journal of Sport Science. 2024 2024;24(5):614-22. PMID: rayyan-14777234. doi: doi:10.1002/ejsc.12080.

176.	Van Oost N, Masci F, Schyvens AM, Peters B, Dirix H, Ross V, et al., editors. A new perspective on validation of wearables for stress monitoring of occupational drivers. 2024 2024: Institute of Electrical and Electronics Engineers Inc.

177.	Vermunicht P, Makayed K, Buyck C, Knaepen L, Piedrahita Giraldo JS, Naessens S, et al. Continuous heart rate measurements in patients with cardiac disease: Device comparison and development of a novel artefact removal procedure. Digit Health. 2025 2025-1;11:20552076251337598. PMID: rayyan-289034246. doi: doi:10.1177/20552076251337598.

178.	Wallen MP, Gomersall SR, Keating SE, Wisløff U, Coombes JS. Accuracy of heart rate watches: Implications for weight management. PLoS ONE. 2016 2016;11(5). PMID: rayyan-14777256. doi: doi:10.1371/journal.pone.0154420.

179.	Walzel S, Mikus R, Rafl-Huttova V, Rozanek M, Bachman TE, Rafl J. Evaluation of Leading Smartwatches for the Detection of Hypoxemia: Comparison to Reference Oximeter. Sensors (Basel). 2023 2023-11-14;23(22). PMID: rayyan-14777257. doi: doi:10.3390/s23229164.

180.	Wang R, Blackburn G, Desai M, Phelan D, Gillinov L, Houghtaling P, et al. Accuracy of Wrist-Worn Heart Rate Monitors. JAMA Cardiology. 2017;2(1):104-6. doi: 10.1001/jamacardio.2016.3340.

181.	Weis A, Leroy M, Jux C, Rupp S, Backhoff D. Oxygen saturation measurement in cyanotic heart disease with the Apple watch. Cardiology in the Young. 2024 2024. PMID: rayyan-289034270. doi: doi:10.1017/S1047951124025216.

182.	Williams K, Jamieson A, Chaturvedi N, Hughes A, Orini M. Validation of Wearable Derived Heart Rate Variability and Oxygen Saturation from the Garmin's Health Snapshot. Mrc Unit for Lifelong Health and Ageing, Institute of Cardiovascular Science, University College London, London, United Kingdom University College London, London, United Kingdom: IEEE Computer Society; 2023 2023. ISBN: 23258861 (ISSN); 9798350382525 (ISBN).

183.	Wolf SM, Seidel P, Ockenga T, Schoder D, Bui TX, editors. Heart-to-Wear: Assessing the Accuracy of Heart Rate Sensor Measurements of Wearable Devices in Uncontrolled Environments. 2024 2024: IEEE Computer Society.

184.	Yang J, Lu Z, Li X, Tao K, Qiu J. Validity of Hera Leto consumer earbuds for heart rate monitoring during stationary cycling. Scientific Reports. 2025 2025;15(1). PMID: rayyan-289034283. doi: doi:10.1038/s41598-025-00559-5.

185.	Yasuda M, Silverman J, Best AM. Can Caregivers Reliably Assess Their Child’s Heart Rate and Respiration Rate Using Smartphone or Smartwatch Applications? Pediatric Emergency Care. 2025 2025. PMID: rayyan-289034284. doi: doi:10.1097/PEC.0000000000003400.

186.	Yi L, Lv ZH, Hu SY, Liu YQ, Yan JB, Zhang H, et al. Validating the accuracy of a multifunctional smartwatch sphygmomanometer to monitor blood pressure. Journal of Geriatric Cardiology. 2022 2022;19(11):843-52. PMID: rayyan-14777287. doi: doi:10.11909/j.issn.1671-5411.2022.11.004.

187.	Yu H, Kotlyar M, Dufresne S, Thuras P, Pakhomov S. Feasibility of Using an Armband Optical Heart Rate Sensor in Naturalistic Environment. 28th Pacific Symposium on Biocomputing, PSB 2023. 2023 2023(2023):43-54. PMID: rayyan-14777288. doi: doi:10.1142/9789811270611_0005.
