# Supplementary material for: The Representation of Different Populations in Studies Assessing the Validity of Consumer Wearable Photoplethysmography-Based Measurements: Scoping Review
Source: JMIR Mhealth Uhealth. 2026 Aug 28;14:e87324. doi: 10.2196/87324 (PMC13524368; doi:10.2196/87324)
Supplement: Supplementary file 5 [file mhealth-v14-e87324-s004.docx]

## Multimedia Appendix 4: Table with distribution of sex, age, weight, height, and BMI per PPG-based measurement

|  | PR | | HVR | | BP | | SpO_2_ | | RR | |
| --- | --- | --- | --- | --- | --- | --- | --- | --- | --- | --- |
|  | N of studies | Value | N of studies | Value | N of studies | Value | N of studies | Value | N of studies | Value |
| **Sex (%women)** |  |  |  |  |  |  |  |  |  |  |
| Mean±SD | 157 | 43±19 | 16 | 45±13 | 7 | 45±5 | 23 | 38±15 | 5 | 47±31 |
| **Age (years)** |  |  |  |  |  |  |  |  |  |  |
| Mean±SD | 132 | 41±11 | 12 | 34±11 | 6 | 45±15 | 16 | 51±13 | 3 | 50±13 |
| Range | 50 | 0.1-94 | 5 | 15-74 | 3 | 18-70 | 8 | 0.1-94 | 1 | 27-74 |
| **Weight (kg)** |  |  |  |  |  |  |  |  |  |  |
| Mean±SD | 85 | 60.9±13.3 | 4 | 78.0±15.4 | 1 | 70.8±13.8 | 9 | 72.2±17.3 | 1 | 94.7±19.4 |
| Range | 27 | 3.8-167.2 | 1 | 60.6-150.4 |  |  | 3 | 3.8-167.2 | 1 | 60.6-150.4 |
| **Height (cm)** |  |  |  |  |  |  |  |  |  |  |
| Mean±SD | 83 | 166±15.9 | 3 | 172±18.5 | 1 | 174±10.0 | 9 | 162±9.0 | 1 | 176±10.2 |
| Range | 25 | 52-198 | 1 | 158-198 |  |  | 3 | 52-198 | 1 | 158-198 |
| **BMI (kg/m2)** |  |  |  |  |  |  |  |  |  |  |
| Mean±SD | 78 | 24.6±4.0 | 5 | 25.8±4.6 | 2 | 28.1±6.3 | 8 | 26.0±5.1 | 3 | 26.7±4.5 |
| Range | 24 | 16.3-52.0 | 1 | 21.4-52.0 |  |  | 5 | 17.3-52.0 | 1 | 21.4-52.0 |
| <18.5 kg/m2 (%) | 78 | 6 | 5 | 5 | 2 | 6 | 8 | 8 | 3 | 3 |
| 18.5-24.9 kg/m2 (%) |  | 50 |  | 43 |  | 23 |  | 33 |  | 34 |
| 25-29.9 kg/m2 (%) |  | 29 |  | 32 |  | 31 |  | 34 |  | 39 |
| >30 kg/m2 (%) |  | 12 |  | 19 |  | 37 |  | 23 |  | 23 |
| **Fitzpatrick scale** |  |  |  |  |  |  |  |  |  |  |
| Mean | 16 | 2.9 | 2 | 2.5 |  |  | 6 | 2.7 | 1 | 2.5 |
| Type I (%) | 16 | 15 | 2 | 18 |  |  | 6 | 14 | 1 | 23 |
| Type II (%) |  | 35 |  | 31 |  |  |  | 37 |  | 49 |
| Type III (%) |  | 24 |  | 20 |  |  |  | 27 |  | 22 |
| Type IV (%) |  | 14 |  | 9 |  |  |  | 12 |  | 0 |
| Type V (%) |  | 5 |  | 9 |  |  |  | 4 |  | 0 |
| Type VI (%) |  | 3 |  | 10 |  |  |  | 3 |  | 0 |
| PR: Pulse rate, HRV: heart rate variability, BP: blood pressure, SpO_2_: peripheral blood oxygen saturation, RR: respiratory rate | | | | | | | | | | |
