# Supplementary material for: The Representation of Different Populations in Studies Assessing the Validity of Consumer Wearable Photoplethysmography-Based Measurements: Scoping Review
Source: JMIR Mhealth Uhealth. 2026 Aug 28;14:e87324. doi: 10.2196/87324 (PMC13524368; doi:10.2196/87324)
Supplement: Supplementary file 6 [file mhealth-v14-e87324-s005.docx]

# Multimedia Appendix 5: Representation of age, BMI, and Fitzpatrick scale categories over time

Figure S2: Percentage of participants in four BMI categories participating in studies assessing the validity of consumer wearable PPG-based measurements per year.

Figure S1: Percentage of adults (18-65 years) and older adults (>65 years) participating in studies assessing the validity of consumer wearable PPG-based measurements per year.

Figure S3: Percentage of participants in the six Fitzpatrick scale categories participating in studies assessing the validity of consumer wearable PPG-based measurements per year.
